# Processing long-distance wh-dependency in the Kyengsang dialect of Korean: an ERP study

**DOI:** 10.3389/fnhum.2026.1774932

**Published:** 2026-04-22

**Authors:** Wonil Chung, Keonwoo Koo

**Affiliations:** Department of English Language and Literature, Dongguk University, Seoul, Republic of Korea

**Keywords:** event-related potentials (ERP), Kyengsang dialect Korean, long-distance wh-dependency, Q-island effects, question particle, wh-in-situ processing

## Abstract

Kyengsang Dialect Korean (KDK) is a wh-in-situ language that morphologically distinguishes content (or wh-) and polar questions via sentence-final question particles (QPs). This study investigates how KDK comprehenders build dependencies between wh-indeterminates and QPs, and how they compute question–answer concord. Two experiments−such as acceptability judgments and event-related potentials (ERPs) − tested sensitivity to feature matching and locality constraints. In the acceptability task, speakers showed robust interactions between QP type and wh-licensing configuration, with additional degradation in island environments. These patterns indicate that dependency resolution is guided by morpho-syntactic agreement while remaining sensitive to structural constraints, even in the absence of overt wh-movement. ERP recordings revealed three dissociable signatures that map onto successive stages of dependency formation. First, a right anterior negativity (RAN) emerged for feature mismatch between a wh-indeterminate’s [+WH] feature and a polar QP, consistent with the rapid detection of illicit licensing. Second, a left anterior negativity (LAN) indexed increased working-memory costs when the [+WH] feature had to be maintained or retrieved across an island boundary. Third, an extended anterior negativity (EXAN) reflected ongoing feature-match monitoring under question–answer discord. Together, the behavioral and neural results suggest that KDK speakers actively maintain and retrieve the [+WH] feature of an in-situ wh-indeterminate to establish syntactically licensed dependencies with matrix QPs, including configurations that challenge locality. Comparisons with Japanese and with wh-fronting languages (e.g., English/German) indicate that KDK engages similar incremental, feature-driven mechanisms for dependency resolution. The findings support a feature-based model in which the parser predicts and matches [+WH] with the appropriate QP at the earliest opportunity, providing neurocognitive evidence that wh-in-situ processing parallels filler–gap computation in wh-movement languages.

## 1 Introduction

### 1.1 Wh-movement and filler-gap dependency

Wh-questions, i.e., interrogatives containing wh-elements such as what and who have long constituted a central topic in linguistic inquiry. They have attracted continued attention not only in theoretical syntax (e.g., Chomsky, 1986, 1995) but also in research on language processing (e.g., Fodor, 1978, 1989; Crain and Fodor, 1985; Stowe, 1986; Frazier and Clifton, 1989; de Vincenzi, 1991; Van der Meulen et al., 2005; Ueno and Kluender, 2009; Jyotishi et al., 2017; Grimaldi S. et al., 2023; Grimaldi M. et al., 2023; Kong and Hsu, 2023; Pagliarini et al., 2025; Lutken and Legendre, 2025). Within the electrophysiological domain, a substantial body of work has examined the neural correlates of wh-question processing using event-related potentials (ERPs) (Kluender and Kutas, 1993a, 1993b; Osterhout et al., 1996; Müller et al., 1997; Kluender and Münte, 1998; Kaan et al., 2000; Fiebach et al., 2001, 2002a, 2002b; Felser et al., 2003; Phillips et al., 2005; Gouvea et al., 2010; Christiansen et al., 2012; Lo and Brennan, 2021; Sayehli et al., 2022; Grimaldi S. et al., 2023; Grimaldi M. et al., 2023; Chacón et al., 2024; Jap and Hsu, 2025). Most of these studies have focused on English and German, languages characterized by wh-movement, in which wh-elements must be fronted to the beginning of the clause.

Consider the examples in (1a-b). In English, a wh-object such as what must be displaced to clause-initial position (except in echo questions, e.g., John drank WHAT?), whereas its corresponding non-wh (in)definite object such as a/the drink remains in its canonical post-verbal position.

(1) a. *Wh-question*:

What did John drink __?

*FILLERGAP.*

b. *Yes/no-question*:

Did John have a/the drink?

In psycholinguistic terminology, the displaced wh-phrase is referred to as a filler, while its canonical position where interpretation is ultimately established is termed the gap. Comprehension depends on the successful formation of this filler–gap dependency. The filler’s grammatical function (e.g., subject, object) and semantic role (e.g., agent, patient) often remain underspecified until the parser encounters the gap, at which point this information can be retrieved and integrated (Fodor, 1989).

Electrophysiological research has consistently associated the processing of filler–gap dependencies with a component known as the left anterior negativity (LAN), a negative-going deflection around 300–500 ms and typically left-lateralized over frontal scalp regions. LAN effects may appear in transient (phasic) form, lasting several hundred milliseconds, or as sustained slow-wave activity persisting for several seconds. Beyond their established links to morphosyntactic and phrase-structure violations (e.g., Neville et al., 1991; Coulson et al., 1998; Martín-Loeches et al., 2005; see Vos et al., 2001, for review), LAN effects have also been associated with the working-memory demands of maintaining and retrieving displaced wh-fillers (Kluender and Kutas, 1993a, 1993b; King and Kutas, 1995; Müller et al., 1997; Kluender and Münte, 1998; Fiebach et al., 2002a, 2002b; Felser et al., 2003; Epstein et al., 2013; Lo and Brennan, 2021; Grimaldi S. et al., 2023; Grimaldi M. et al., 2023; Nakamura and Rossi, 2025). For instance, Kluender and Kutas (1993a, 1993b) observed phasic LAN responses immediately following both the filler and the gap in English object wh-questions, suggesting that filler storage and subsequent retrieval are indexed by distinct LAN processes. Similarly, King and Kutas (1995) reported a sustained frontal negativity between the filler and the gap, as well as a phasic LAN effect following the gap, during the comprehension of English object-relative clauses.

More recent ERP research has identified P600 effects either instead of or alongside LAN effects at or near the gap position. The P600 is a broadly distributed positive deflection, peaking approximately 500–800 ms after stimulus onset and maximal over centro-posterior scalp sites. It is generally interpreted as reflecting syntactic reanalysis or integration difficulty. Kaan et al. (2000), for example, reported a P600 at the verb preceding the gap [e.g., drink in (1a)] in wh-questions relative to yes/no questions, interpreting this positivity as an index of the increased effort required to integrate the wh-filler into the evolving syntactic structure (see also Fiebach et al., 2002a, 2002b; Ueno and Kluender, 2003; Phillips et al., 2005; Gouvea et al., 2010; Grimaldi S. et al., 2023; Grimaldi M. et al., 2023; Christiansen et al., 2012; Jap and Hsu, 2025). Other studies have reported co-occurring LAN and P600 components in wh-question processing across several languages, including English (Phillips et al., 2005), German (Fiebach et al., 2002a, 2002b; Felser et al., 2003), and even in “scrambled” wh-questions in Japanese (Ueno and Kluender, 2003).

### 1.2 Standard Korean and the Kyengsang dialect of Korean: a wh-in-situ language

Unlike English and German, Standard Korean (SK) does not formally distinguish between a wh-interpreted element and an indefinite; instead, both are realized by the same form, commonly referred to as an indeterminate element. Although such elements can receive a wh-interpretation, they typically remain *in situ* at the surface level, which is why SK is classified as a wh-in-situ language. While overt movement of these elements is possible, such movement is considered a case of scrambling rather than wh-movement in the sense observed in languages like English and German.

A further distinction between SK and languages such as English and German lies in the use of an overt question/interrogative particle (QP), which functions as a complementizer. In SK, this particle, typically realized as -*ni* in matrix clauses, serves as a morphosyntactic marker that signals whether the clause is interpreted as a wh-question or a polar/yes-no question. Specifically, when the QP is accompanied by falling intonation, the clause is interpreted as a wh-question, whereas rising intonation marks the clause as a polar question.

The Kyengsang Dialect of Korean (KDK), spoken in the southern provinces of Korea, shares the same grammatical mechanisms as SK in question formation. However, it diverges from SK in that it distinguishes two types of QPs morphologically, rather than through intonation. Specifically, the particle -*na* marks polar questions, whereas -*no* is used to form information-seeking wh-questions. This distinction renders KDK a mirror image of English in wh-question formation. In KDK, a wh-question is identified through the presence of a [+WH] QP, whereas in English, it is marked by the overt movement of the wh-element.

In addition to signaling the type of question, the QP in KDK (as well as in SK) plays a crucial role in determining the scope of a wh-indeterminate, that is, an indeterminate element interpreted as a wh-expression. The scope of such an element corresponds to the domain of the clause that is being questioned. In wh-movement languages such as English and German, the overt position to which a wh-phrase moves overtly indicates its scope within the sentence. In contrast, in KDK (and SK), it is the position of the QP, rather than that of the wh-indeterminate itself, that determines the interpretive scope of the wh-expression.

From a syntactic perspective, Huang (1982) and subsequent researchers have proposed that, analogous to overt wh-movement in English and German, a wh-in-situ element undergoes covert movement to the corresponding QP at the level of Logical Form (LF), where it receives its scope interpretation in wh-in-situ languages such as Chinese, Japanese, and Korean. Alternatively, Tsai (1994), Chung (1996), and others have argued that the relationship between a wh-in-situ element and its associated QP is not established via covert movement but rather through Q-binding (cf. Baker, 1970), a binding-theoretic relation that determines the wh-scope without syntactic displacement.

### 1.3 Processing of KDK wh-questions

In keeping with syntactic considerations of wh-indeterminates (i.e., indeterminate elements interpreted as wh-expressions), this paper moves on to examine how speakers of KDK resolve long-distance wh-dependencies during sentence processing; specifically, how they establish the relation between a wh-indeterminate and the [+WH] QP in determining the scope of the wh-indeterminate. As discussed above, KDK distinguishes yes–no questions and information-seeking wh-questions through distinct sentence-final QPs: -*na* marks polar questions, while -*no* signals information-seeking wh-questions. When encountering an indeterminate expression such as *mwues* (‘what/something’), which can function either as a wh-element or as an indefinite in sentences like (2), KDK speakers may interpret it in one of three ways: as a [+WH] wh-expression, as a [–WH] indefinite, or as an underspecified [+/-WH] ambiguous form.

(2) a. Cheli-nunmwues-ulmasi-ess-no?

Cheli-TOPanything/what-ACCdrink-PST-[+WH] QP.

‘What kind of drink did Cheli have?’

b. Cheli-nunmwues-ulmasi-ess-na?

Cheli-TOPanything/what-ACCdrink-PST-Polar QP.

‘Did Cheli have any kind of drink?’

If KDK speakers recognize *mwues* as a [+WH] wh-expression, sentences like (2b) containing a subsequent sentence-final polar/[–WH] QP (i.e., −*na*) are expected to be judged as less acceptable than those like (2a) with a [+WH] QP (i.e., −*no*).

Furthermore, based on the widely accepted view (e.g., Huang, 1982; Tsai, 1994; Chung, 1996) that in KDK, an indeterminate recognized as [+WH] (i.e., a wh-element) integrates or feature-matches with the [+WH] interrogative particle (QP) in a manner parallel to the integration between a wh-filler (i.e., an overly moved wh-element) and its corresponding gap in English, we hypothesize that the integration or feature-matching of a [+WH] indeterminate with a polar QP will elicit a neural signature (such as enhanced LAN or P600) indicative of a failed integration or feature-matching process, analogous to that observed in filler–gap dependency violations. By contrast, when the indeterminate is interpreted as [–WH] or as underspecified ([+/-WH]), its combination with the [–WH] QP is not expected to evoke such a neural response or may yield a substantially attenuated effect.

A question arises as to how an embedded indeterminate behaves when it co-occurs with either the declarative [−Q] complementizer -*tako* or the interrogative [+Q] particle -*nunci*? The embedded clause is then followed by a matrix QP, either [+WH] or polar. Examples (3a–d) illustrate these patterns.

(3) a. Cheli-nun[nay-ka mwues-ulmasi-ess-**tako**].

Cheli-TOPI-NOM anything/what-ACC drink-PST-COMP_[−Q]_.

al-ko iss-**no**?

know-Prog-Polar QP[+WH].

‘What kind of drink does Cheli know that I had?’

b. Cheli-nun[nay-ka mwues-ulmasi-ess-**tako**].

Cheli-TOPI-NOM anything/what-ACC drink-PST-COMP_[−Q]_.

al-ko iss-**na**?

know-Prog-Polar QP.

‘Does Cheli know that I had any kind of drink?’

c. Cheli-nun[nay-ka mwues-ulmasi-ess-**nunci**].

Cheli-TOPI-NOM anything/what-ACC drink-PST-COMP_[+Q]_.

al-ko iss-**no**?

know-Prog-QP[+WH].

‘Does Cheli know what kind of drink I had?Or */? What kind drink does Cheli know whether I had?’.

d. Cheli-nun[nay-ka mwues-ulmasi-ess-**nunci**].

Cheli-TOPI-NOM anything/what-ACC drink-PST-COMP_[+Q]_.

al-ko iss-**na**?

know-Prog-Polar QP.

‘Does Cheli know what kind of drink I had?’

The examples in (3a) and (3b) share the same structural configuration as those in (2a) and (2b), except that the former contain an indeterminate element in the embedded clause, whereas the latter contain one in the matrix clause. Accordingly, the same logic that applies to (2a) and (2b) also extends to (3a) and (3b). KDK speakers initially analyze the embedded *mwues* as a [+WH] wh-expression. On this basis, sentences such as (3b), which end with a matrix polar/[–WH] QP (i.e., −*na*), are predicted to be judged less acceptable than those like (3a) that end with a matrix [+WH] QP (i.e., −*no*). If the wh-dependency between a wh-indeterminate and a [+WH] QP is established via integration (Gibson, 1998, among others) or Q-indexing (Baker, 1970, among others), the embedded [+WH] indeterminate fails to integrate or feature-match with the QP in the former, whereas no such failure arises in the latter. This prediction is borne out by the experiments reported below.

We now turn to the predictions for (3c) and (3d), where the embedded clause ends with a [+Q] QP. Given that an indeterminate is, by default, interpreted as a [+WH] element, in both (3c) and (3d) the embedded wh-indeterminate readily establishes a local dependency with the embedded [+Q] particle. As a result, the matrix [+WH] QP in (3c) cannot form a dependency with the same wh-indeterminate, which in turn fails to take matrix scope: an effect parallel to a wh-island violation in English (or any other overt movement language).^1^ Thus, the configuration in (3c) is predicted to receive substantially lower acceptability ratings than the sentence (3d) with the matrix polar QP. Given the wh-dependency formation via integration or Q-indexing, we further predict that an unlicensed matrix [+WH] QP, as in (3c), will elicit ERP effects (e.g., an enhanced LAN or P600) typically associated with integration or feature-matching failure, relative to a properly licensed matrix [+WH] QP, as in (3a).

According to our hypothesis, (3d) constitutes an embedded wh-question within a matrix polar question. In this structure, the wh-indeterminate *mwues* ‘what’ is locally licensed by the embedded complementizer –*nunci*. As a result, the matrix clause forms a polar question, asking whether Cheli knows the answer. Accordingly, the sentence can be naturally answered with a positive polarity particle such as *ung* ‘yes’, followed by a verbal complex, as illustrated in (4b). In contrast, (3c) involves a matrix [+WH] QP –*no*. If the embedded wh-indeterminate were locally associated with the embedded QP, the matrix [+WH] QP would be left without a proper licenser, resulting in an anomalous interpretation. In principle, the sentences could instead be interpreted as a matrix wh-question if the embedded wh-indeterminate bypasses the embedded QP and associates with the matrix [+WH] QP. However, this interpretation incurs a substantial cognitive cost, as it requires circumventing the locality constraint that normally requires a wh-indeterminate to associate with the closest QP. Under this non-local dependency, the sentence may be answered with a fragment such as *khola-lul* ‘*coke’*, as shown in (4a).

(4) a. khola-lul.

Coke-ACC.

‘A coke.’

b. ung, al-ko iss-e.

yes, know-Prog-Informal.

‘Yes, I know.’

### 1.4 Research questions

Building on previous theoretical and electrophysiological studies of wh-dependencies and question formation, this study investigates how speakers of KDK process long-distance dependencies involving wh-indeterminate elements and Q-particles (QPs). In particular, it examines how KDK speakers determine the scope of wh-indeterminates during real-time sentence processing and how this process interacts with the language’s morphosyntactic features.

First, the study examines how an indeterminate is interpreted—whether as [+WH], [–WH], or underspecified—and how this interpretation affects dependency formation and scope. A mismatch between a [+WH] indeterminate and a polar QP is predicted to reduce acceptability and elicit ERP effects (e.g., enhanced LAN or P600), whereas feature matching should incur no such cost.

Second, the study investigates the processing of long-distance wh-dependencies in embedded clauses. Specifically, it tests sentences in which an embedded indeterminate occurs with either the declarative [−Q] particle (−*tako*) or the interrogative [+Q] particle (−*nunci*), followed by a matrix [+WH] or polar QP. This 2 × 2 design examines whether an embedded [+Q] particle blocks long-distance wh-association with the matrix QP, yielding a Q-island effect.

Third, the study explores the processing and acceptability consequences of locality violations. If a wh-indeterminate fails to associate locally with a [+Q] particle and instead attempts a long-distance dependency, the sentence is expected to show increased processing difficulty and reduced acceptability, suggesting that wh-dependency formation in KDK is constrained by locality principles.

## 2 Experiment 1: Acceptability rating tasks

The acceptability rating tasks were designed to determine whether KDK speakers are sensitive to two potential sources of unacceptability: (i) mismatched dependencies between wh-indeterminates and sentence-final polar QPs, as compared to matched dependencies with [+WH] QPs, and (ii) misalignments in question answer concord (QAC), which may impose greater processing demands and reduce sentence acceptability. To this end, two separate acceptability rating experiments were conducted. The first manipulated the dependency between wh-indeterminates (Nonisland vs. Island) and the type of sentence-final QP (Wh vs. Polar), following the structural configurations as in (3a-d). The lexical items across the four conditions were identical, except for the embedded complementizer and sentence-final Q-particle. The second examined dialogue consists of a question paired with either a fragment answer (FA) or a positive polar answer (PPA: *ung* ‘yes’), with QAC systematically varied (FA vs. PPA), as in (4a-b) as responses to (3a-d). Because our focus was on question formation, we employed only felicitous responses across the four question conditions [i.e., responses in which the answers matched the question particles (QPs)].^2^

### 2.1 Participants

Forty native speakers of Korean (mean age = 23 years, range = 20–29; 21 female) participated as paid volunteers. All participants were lifelong residents (19–26 years) of regions in South Kyengsang Province where KDK is the dominant dialect. They did not participate in the ERP study and gave written informed consent before participating.

### 2.2 Materials and procedure

We conducted two acceptability-rating experiments. The two tasks were conducted as two separate acceptability-rating experiments. Participants completed the first experiment before proceeding to the second experiment. The first experiment employed a 2 × 2 factorial design crossing Structure type (Nonisland vs. Island) with Question Particle (QP) type (Wh vs. Polar), as illustrated in the question portion of Table 1. Twenty-four sets of question items were constructed according to this design (see Table 1 for sample stimuli). Each item was presented in a pencil-and-paper format, and participants rated its acceptability on a five-point Likert scale (1 = highly unacceptable, 5 = highly acceptable). The 24 experimental sets were distributed across four counterbalanced lists following a Latin square design, such that each participant rated six items per condition. Each list additionally contained 12 filler questions that were structurally comparable to the experimental items. Specifically, the fillers included biclausal structures with a relative clause and a clausal-final question particle, mirroring the overall syntactic configuration of the target sentences (e.g., *학원에서 누구를 가르쳤던 선생님이 떠났나/떠났노*?(*hakweneyse nwukwulul kaluchyessten sensayngnimi ttenassna/ttenassno*?) ‘Did the teacher who had taught {someone/who} at the academy leave?’).

**Table 1:** Examples of experimental materials.

Indeterminate	Q particle	Question	Answer	Answer type
Nonisland	WH **(I)**	**Nonisland + WH QP (−tako([−Q]) + −no([+WH])** Q: 니는 /영이가 /무신 책을/ 읽었**다고/ 알고 있노**? Ni-nun [Yengi-ka mwusin chayk-ul **ilk-ess-ta-ko**] **alko iss-no**? you-Top Y.-Nom what book-Acc **read-Pst-Dec-C**know-**Q**_**[+WH]**_ ‘What book do you know that Yenghi read?’	A: 소설책을. **Soselchayk-ul.** novel-Acc ‘A novel ‘	FA
	Polar **(II)**	**Nonisland + Polar QP (−tako([−Q]) + −na(Polar)** Q: 니는 영이가 무신 책을 읽었**다고 알고 있나**? Ni-nun [Yengi-ka mwusin chayk-ul **ilk-ess-ta-ko**] **alko iss-na**? you-Top Y.-Nom what book-Acc **read-Pst-Dec-C**know-**Q**_**[Polar]**_ ‘Do you know that Yenghi read what kind of book?’	A: 응, 알고 있어. **Ung, alko iss-e** Yes, know-Dec ‘Yes, I do.’	PPA
Island	WH **(III)**	**Island + WH QP (−nunci([+Q]) + −no([+WH])** Q: 니는 영이가 무신 책을 읽었**는지 알고 있노**? Ni-nun [Yengi-ka mwusin chayk-ul **ilk-ess-nunci**] **alko iss-no**? you-Top Y.-Nom what book-Acc **read-Pst-Q**know-**Q**_**[+WH]**_ ‘Do you know what kind of book Yenghi read? Or */? What kind drink do you know whether Yenghi read?’	A: 소설책을. **Soselchayk-ul.** novel-Acc ‘A novel ‘	FA
	Polar **(IV)**	**Island + Polar QP (−nunci([+Q]) + −na(Polar)** Q: 니는 영이가 무신 책을 읽었**는지 알고 있나**? Ni-nun [Yengi-ka mwusin chayk-ul **ilk-ess-nunci**] **alko iss-na**? you-Top Y.-Nom what book-Acc **read-Pst-Q**know-**Q**_**[Polar]**_ ‘Do you know what kind of book Yenghi read?’	A: 응, 알고 있어. **Ung, alko iss-e.** Yes, know-Dec ‘Yes, I do.’	PPA

Each item consists of a subject NP, an indeterminate-containing embedded clause either with a declarative or an interrogative Comp, and a [+/-WH] Q-particle in the sentence-final matrix clause. The four conditions (I)-(IV) used in Experiments 1 and 2 are each characterized as Nonisland-WH, Nonisland-Polar, Island-WH, Island-Polar, where Island/Nonisland represents the structure of the embedded clause, and WH/Polar represents the type of Q-particle. Answers to the preceding question are characterized as concordant in relation to either type of Q-particle in the latter’s matrix clause. Critical regions are highlighted.

Immediately following this task, participants completed a second acceptability-rating experiment that used a 2 (Structure: Nonisland vs. Island) × 2 (Response: FA vs. PPA) design but embedded the target questions and corresponding answers within short dialogues (see Table 1, question and response portions). In each dialogue, Speaker A produced either a wh- or a polar question, and Speaker B responded with either a nominal-phrase (NP) fragment answer or a yes/no answer corresponding to the [+WH]/polar QP. The answer type served as a diagnostic for the interpretation of the indeterminate by examining whether participants preferred fragment answers (consistent with a matrix *wh* interpretation) or positive polar responses (consistent with an embedded polar interpretation). Participants evaluated each dialogue on the same five-point scale used in the first task. None of the questions appearing in the dialogue were repeated from the first experiment. The 24 dialogue sets were likewise distributed across four Latin square lists (six items per condition), each supplemented by 12 filler dialogues matched in structural complexity. Across both experiments, lexical content was fully controlled: all words were held constant except for the alternation of matrix QPs (*−no* vs. –*na*) and embedded complementizers (declarative –*tako* vs. interrogative –*nunci*).

### 2.3 Results of acceptability rating tasks

We analyzed the acceptability ratings using linear mixed-effects models (LMMs) implemented in R (version 4.0.2), employing the *lme4* package (Bates et al., 2015) and *lmerTest* (Kuznetsova et al., 2017). Prior to analysis, acceptability ratings were z-transformed within participants to control for individual differences in scale use, such that each participant’s ratings were standardized to a mean of zero and a standard deviation of one. The models included fixed effects of Structure, QP, and their interaction (Structure × QP), along with random slopes and intercepts for both participants and items. The categorical predictors were contrast-coded: for Structure, Island = +0.5 and Nonisland = −0.5 in both tasks; for QP, [+Wh] = +0.5 and Polar = −0.5 in the first task, and Fragment Answer = +0.5 and Polar Answer = −0.5 in the second. When significant main effects or interactions emerged, we conducted *post hoc* pairwise comparisons using the *emmeans* package (Lenth, 2021), applying Holm correction for multiple comparisons.

#### 2.3.1 Question-based acceptability task

**Figure 1:**
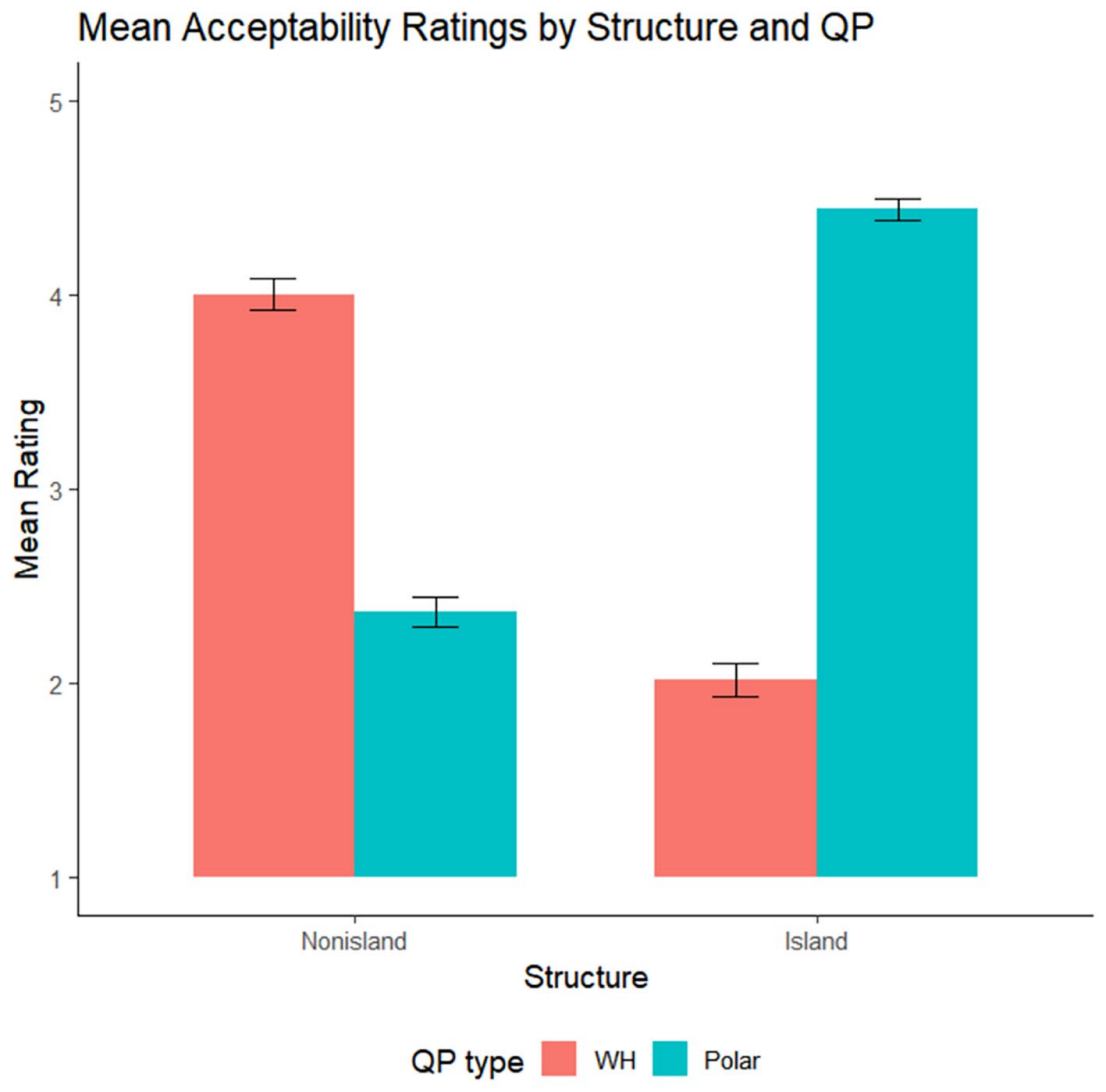
Mean acceptability ratings by structure and QP type in the acceptability rating task based on questions. Error bars represent ±1 SE. Raw mean scores are shown for ease of interpretation; all inferential statistical analyses were conducted on z-transformed ratings.

In the first task, as shown in Figure 1 and Table 2, participants rated Nonisland–Wh sentences (M = 4.00) as more acceptable than Nonisland–Polar sentences (M = 2.37), while Island–Polar sentences (M = 4.45) were judged more acceptable than Island–Wh sentences (M = 2.01).^3^ As summarized in Table 3, Structure showed no significant main effect, whereas QP was significant (Estimate = −0.21, SE = 0.092, *t* = −2.23, *p* < 0.05), reflecting the overall preference for Polar over Wh sentences. Crucially, the Structure × QP interaction reached significance (Estimate = −2.80, SE = 0.176, *t* = −15.95, *p* < 0.001), indicating that the relative acceptability of *Wh* versus Polar questions depended on the structural context.

**Table 2:** Mean acceptability with standard deviation in parentheses in the acceptability rating task based on a question on a scale from 1 (unacceptable) to 5 (acceptable).

Matrix Q-particle	Indeterminate location	
	Nonisland	Island
WH	4.00 (1.25)	2.01 (1.34)
Polar	2.37 (1.25)	4.45 (0.88)

**Table 3:** The results of the linear mixed model in the acceptability task based on the question.

Fixed factors	Estimate	SE	df	*t*-value	*p*-value
(Intercept)	0.005	0.028	21.2	0.169	>0.1
Structure	0.077	0.068	38.9	1.124	>0.1
QP	−0.206	0.092	41.4	−2.234	< 0.05*
Structure* QP	−2.804	0.176	42.9	−15.945	< 0.001***
lmer(score_z ~ Structure * QP + (1 + Structure*QP |subject) + (1 + Structure*QP | item)					

Pairwise follow-up analyses revealed that within Wh-question contexts (−*no*), Structure had a significant effect (Estimate = 1.33, SE = 0.135, *t* = 9.85, *p* < 0.001), with Nonisland sentences receiving higher ratings than Island sentences. Conversely, within Polar questions (−*na*), Structure also yielded a significant effect (Estimate = −1.48, SE = 0.091, *t* = −16.31, *p* < 0.001), but in the opposite direction—Island sentences were rated higher than Nonisland ones. Moreover, within the Island condition (−*no* vs. –*na*), QP showed a significant effect (Estimate = 1.61, SE = 0.122, *t* = 13.16, *p* < 0.001), with Polar sentences (−*na*) preferred over Wh sentences (−*no*); in contrast, within the Nonisland condition, QP was again significant (Estimate = −1.20, SE = 0.138, *t* = −8.66, *p* < 0.001), but in the reverse direction—Wh sentences were rated more acceptable than Polar sentences.

#### 2.3.2 Dialogue-based acceptability task

**Figure 2:**
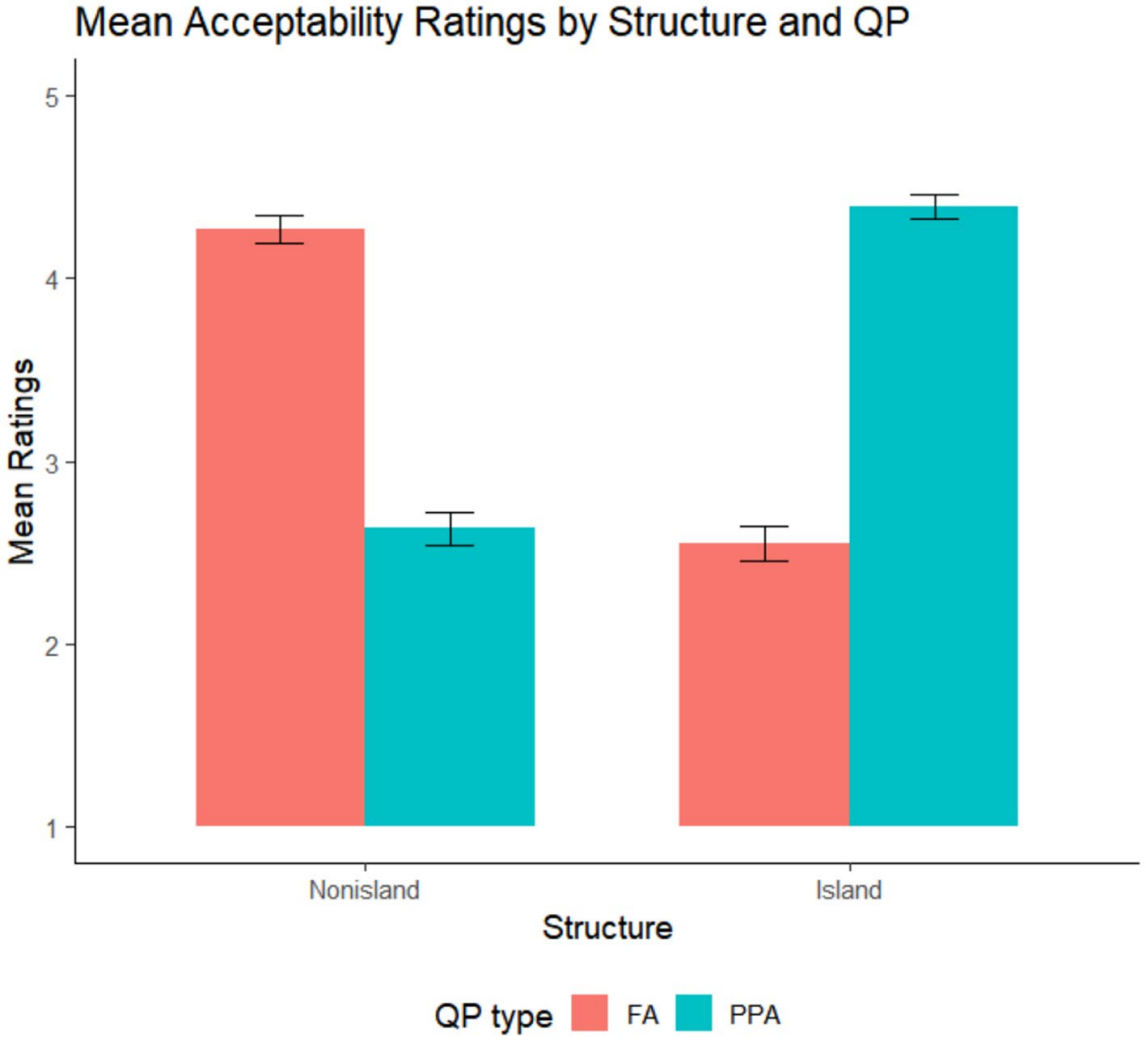
Mean acceptability ratings by structure and QP type in the acceptability rating task based on dialogues. Error bars represent ±1 SE. Raw mean scores are shown for ease of interpretation; all inferential statistical analyses were conducted on z-transformed ratings.

In the second task, which involved question–answer dialogues, the results closely mirrored those of the first experiment. As shown in Figure 2 and Table 4, dialogues containing polar answers to Island-Polar QP questions ([Island-Polar]-PPA) received higher mean ratings (M = 4.39) than those with fragment answers ([Island-Polar]-FA) (M = 2.55). Conversely, for Nonisland questions, fragment answers ([Nonisland-Wh]-FA) (M = 4.27) were judged more acceptable than polar answers ([Nonisland-Polar]-PPA) (M = 2.63).

**Table 4:** Mean acceptability and standard deviation (in parentheses) in acceptability rating task based on dialogues on a scale from 1 (unacceptable) to 5 (acceptable).

Answer	Indeterminate location	
	Nonisland	Island
Fragment	4.27 (1.14)	2.55 (1.49)
Polar	2.63 (1.40)	4. 39 (0.98)

As reported in Table 5, there were no significant main effects of Structure or QP, but the Structure × Response interaction was highly significant (Estimate = −2.34, SE = 0.203, *t* = −11.53, *p* < 0.001), reflecting a crossover pattern between Island and Nonisland structures depending on answer type.

**Table 5:** The results of the Linear Mixed Model in the acceptability task based on dialogue.

Fixed factors	Estimate	SE	df	t-value	*p*-value
(Intercept)	−0.0001	0.029	23.8	−0.006	>0.1
Structure	0.037	0.071	39.0	0.527	>0.1
Response	−0.026	0.105	41.2	−0.248	>0.1
Structure * Response	−2.343	0.203	40.7	−11.528	< 0.001***
lmer(score_z ~ Structure * QP + (1 + Structure*QP |subject) + (1 + Structure*QP | item)					

Pairwise follow-up analyses clarified the source of this interaction. Within Fragment Answer conditions, Structure had a significant effect (Estimate = 1.13, SE = 0.136, *t* = 8.34, *p* < 0.001), with Nonisland dialogues ([Nonisland-Wh]-FA) rated higher than Island ones ([Island-Wh]-FA). In PPA conditions, Structure also showed a significant effect (Estimate = −1.21, SE = 0.111, *t* = −10.91, *p* < 0.001), but again in the opposite direction—Island dialogues ([Island-Polar]-PPA) were judged more acceptable than Nonisland ones ([Nonisland-Polar]-PPA). Additionally, within the Island condition, Response was significant (Estimate = 1.20, SE = 0.146, *t* = 8.18, *p* < 0.001), showing a preference for PPAs over FAs. In contrast, within the Nonisland condition, Response also reached significance (Estimate = −1.15, SE = 0.146, *t* = −7.84, *p* < 0.001), but with the reverse pattern—FAs were judged more acceptable than PPAs.

These results together demonstrate a consistent interaction between structure and question type, showing that island constraints modulate acceptability in both wh- and polar-question contexts in KDK.

### 2.4 Discussion

We first report the offline behavioral results from Experiment 1. Two main findings emerged. First, indeterminate elements in KDK question sentences are by default interpreted as information-seeking wh-elements rather than as indefinites. Second, the embedded interrogative complementizer –*nunci* forms a Q-island, thereby blocking the embedded indeterminate from associating with the matrix [+WH] QP.

The significant degradation in acceptability ratings between Conditions I & II and III & IV provides empirical support for these conclusions, suggesting that the underlying structural constraints remain robust even when participants have ample time for reflection. Overall, participants’ judgments demonstrate that indeterminate elements in KDK questions are initially construed as wh-elements rather than as indefinites. The degraded acceptability observed in the (embedded) Nonisland–(matrix) Polar condition (Condition II; M = 2.37 (out of 5)) indicates that when an indeterminate inside an embedded declarative clause co-occurs with the matrix [–WH] QP, the indeterminate—being preferentially parsed as a wh-element—fails to integrate or establish a feature-matching relation with the polar QP.

A similar pattern arises in the (embedded) Island–(matrix) Wh condition (Condition III; M = 2.01(out of 5)). Here, the indeterminate within an embedded question clause occurs with the matrix [+WH] QP. Because the indeterminate is preferentially interpreted as a wh-element, it immediately enters into a feature-matching relation with the embedded interrogative complementizer. Consequently, the matrix [+WH] QP fails to be properly licensed. This degradedness further suggests that Q-island effects are operative in KDK: since the embedded QP forms a Q-island, the embedded object wh-indeterminate cannot cross it to associate with the matrix [+WH] QP and take matrix scope.

By contrast, the Island–Polar condition [Condition IV; M = 4.45 (out of 5)] exhibited markedly higher acceptability ratings. In this configuration, the embedded wh-indeterminate readily associates with the embedded interrogative complementizer, while the matrix polar QP remains well-formed despite the absence of any feature-matching relation between the two. Finally, the Nonisland–Wh condition [Condition I; M = 4.0 (out of 5)] served as the baseline: here, the wh-indeterminate in the embedded declarative clause legitimately associates with the matrix [+WH] QP, yielding full acceptability, as predicted.

In the second dialogue-based question–answer acceptability task, the rating patterns closely parallel those in the first question-based acceptability task. Dialogues featuring positive polarity answers (PPAs) to embedded island questions are rated more acceptable than their fragment-answer counterparts, whereas fragment answers are preferred over PPAs in embedded Nonisland contexts (Figure 2 and Table 4). Overall, there are significant main effects of both indeterminate type and QP type, as well as a significant interaction between them. This interaction reflects the particularly low acceptability of fragment answers in embedded island contexts compared to the high acceptability of PPAs in the same context, replicating the pattern observed in the first task. Within the fragment-answer condition, nonisland sentences are rated higher than Island sentences, while within the PPA condition, the pattern reverses, with Island sentences receiving higher ratings than nonisland sentences. The second task further confirms that indeterminate elements are, by default, parsed as wh-expressions, and that the embedded interrogative complementizer establishes a local dependency with an embedded wh-indeterminate, thus blocking scope extension to the matrix QP.

Taken together, the offline behavioral acceptability ratings across the four conditions in the first and the second tasks reveal several points of theoretical significance that are in keeping with previous accounts of filler–gap dependency formation. First, indeterminate elements in KDK are not processed as [–WH] or underspecified ([±WH]) elements, but rather as inherently [+WH] elements, interpreted preemptively as wh-expressions during sentence processing. Second, given this inherent [+WH] specification, the relation between wh-indeterminates and QPs (including the embedded interrogative complementizer *-nunci*) in KDK is directly parallel to the filler-gap dependency observed in English and other wh-fronting languages, in line with Ueno and Kluender (2003, 2009). Third, unlike in standard Korean and Japanese—where [+WH] and polar QPs are not morphologically distinguished (Ueno and Kluender, 2003, 2009; Kim and Goodhall, 2016; Cho, 2017)—KDK overtly marks this distinction. This morphological transparency provides compelling evidence that the presence of a local embedded interrogative complementizer blocks an embedded wh-indeterminate from associating with a higher, matrix-level QP, thereby demonstrating that the Q-island constraint is robustly active in KDK.

## 3 Experiment 2: An ERP study

Building on the findings from the acceptability rating tasks, Experiment 2 investigated the neural correlates of scope dependencies and question–answer concordance in KDK. The behavioral data revealed crossover interaction patterns: in Island contexts, polar questions and answers were judged more acceptable than their wh/fragment counterparts, whereas in Nonisland contexts, the pattern was reversed, with wh/fragment questions and answers receiving higher ratings than polar ones. Although main effects of Structure and QP were not consistently observed across tasks, the robust Structure × QP interaction underscored the importance of structural context in determining acceptability. Given that these patterns suggested sensitivity to integration/feature matching between indeterminates and Q-particles, the ERP experiment was designed to identify the neural mechanisms underlying morpho-syntactic dependency formation and to detect potential integration and feature-matching failures during real-time sentence processing.

### 3.1 Participants

Twenty-six native speakers of Korean from South Kyengsang Province participated in the ERP experiment. None had an immersive experience learning English in an English-speaking country. Data from two participants were excluded due to excessive EEG artifacts, resulting in a final sample of 24 participants (mean age = 23 years, range = 19–28; 13 male). All participants were right-handed college students with normal or corrected-to-normal vision and no reported neurological or psychiatric disorders. Written informed consent was obtained from all participants before the experiment, and they received monetary compensation for their participation. Ethical approval for the study was granted by the Dongguk University Institutional Review Board (DUIRB-202106-04).

### 3.2 Materials

The ERP experiment materials were constructed using the same 2 × 2 factorial design as the two acceptability-rating tasks in Experiment 1, crossing Structure type (Nonisland vs. Island) and QP type (Wh vs. Polar). The stimuli consisted of question–answer dialogues. In each dialogue, the question sentence instantiated one of the four experimental conditions, and the subsequent answer was either a nominal-phrase (NP) fragment response or a yes/no response, corresponding to the [+WH] or polar QP, respectively.

A total of 480 experimental dialogues were created and distributed across four counterbalanced lists using a Latin Square design, such that each list contained 30 dialogues per condition for each list (120 experimental trials per list). To reduce predictability and discourage strategic processing, two types of fillers were included. First, 120 filler dialogues were constructed in the same dialogue format, containing responses that were contextually inappropriate given the preceding question [e.g., Q: *니는 영희가 무신 책을 읽었다고 알고 있나*? A: *소설책을 (Q: ninun Yenghuyka mwusin chaykul ilkesstako alko issna*? A: *soselchaykul***)**. ‘Q: Do you know that Yenghi read what kind of book? A: A novel.’]. Second, 240 additional fillers structurally matched to the experimental items in terms of clause structure, length, and the presence of clause-final QPs [e.g., *학원에서 누구를 가르쳤던 선생님이 떠났나/떠났노*?(*hakweneyse nwukwulul kaluchyessten sensayngnimi ttenassna/ttenassno*?) ‘Did the teacher who had taught someone at the academy leave?’], but did not manipulate the critical experimental factors. Each participant viewed one list, which was divided into three experimental blocks to minimize fatigue and maintain attention.

### 3.3 Procedure

Participants were seated in a dimly lit, sound-attenuated booth, where they silently read each sentence and judged whether it “made sense.” The experimental stimuli were presented using a rapid serial visual presentation (RSVP) paradigm implemented in E-Prime (Psychology Software Tools, Inc.).

**Figure 3:**
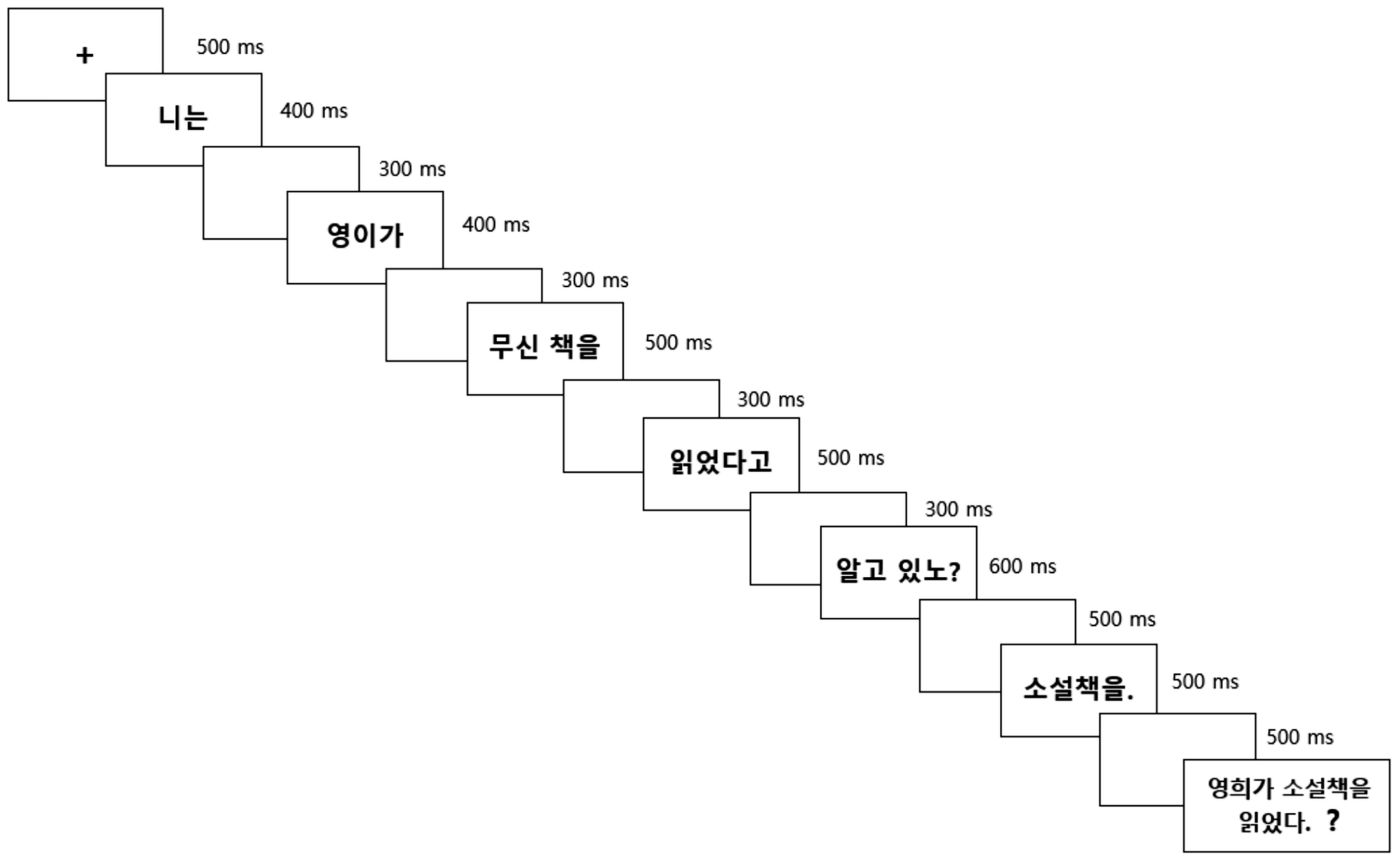
Experimental procedure: example of the Nonisland WH-FA condition.

Each trial began with a 500 ms fixation cross, followed by the sentence presented region by region in the center of the screen (see Table 1). The question regions were displayed for 400–500 ms, depending on the number of syllables, with a 300 ms inter-stimulus interval (ISI). The three critical regions (embedded complementizer, sentence-final QP, and answer) were shown for 500 ms when they consisted of a single four-syllable word (e.g., *soselchayk-ul*), or 600 ms when consisting of two words containing four or five syllables (e.g., *alko iss-na/-no* or *ung, alko iss-e*). A 500 ms interval separated the question from its corresponding answer (see Figure 3).

Following each fragment or yes/no answer, a 500 ms blank screen was presented. Participants then completed a comprehension probe [e.g., *영희가 소설 책을 읽었다* (*Yenghuyka sosel chaykul ilkessta***)** ‘Yenghi read a novel’] to ensure sustained attention throughout the task. They indicated whether the probe sentence was correct or incorrect by pressing one of two buttons (“YES” or “NO”) on a response box (see Figure 3).

Before the main experiment, participants completed 12 practice trials to become familiar with the procedure. The main session consisted of three experimental blocks, with short rest breaks provided between blocks.

### 3.4 EEG recordings

EEG data were continuously recorded from 30 scalp electrodes (Fz, FCz, Cz, CPz, Pz, Oz, FP1/2, F3/4, F7/8, FC3/4, FT7/8, C3/4, T7/8, CP3/4, TP7/8, P3/4, P7/8, O1/2) mounted on a Neuroscan Quik-Cap (USA) and referenced to the linked mastoids. To monitor eye movements and blinks, two pairs of electro-oculogram (EOG) electrodes were positioned above and below the left eye and at the outer canthi of both eyes.

Electrode impedances were kept below 5 kΩ throughout the recording. The EEG signal was amplified with a 0.3–100 Hz bandpass filter and digitized at a 1 kHz sampling rate using a SynAmps2 amplifier (Compumedics Neuroscan, USA).

### 3.5 Data analysis

Trials containing excessive ocular or muscular artifacts were excluded following visual inspection, resulting in an average rejection rate of 9.3%. Only artifact-free trials were included in the ERP averages. The ERPs were time-locked to the onset of the critical regions and averaged over 1,100 ms epochs, comprising a 100 ms pre-stimulus baseline and a 1,000 ms post-stimulus interval. The grand-average waveforms were subsequently low-pass filtered at 30 Hz.

For statistical analyses, wh-dependency effects were examined at three critical regions (embedded complementizer, sentence-final QP, and answer). Electrodes were grouped into six regions of interest (ROIs): Left anterior (F3, FC3); Midline anterior (Fz, FCz); Right anterior (F4, FC4); Left posterior (CP3, P3); Midline posterior (CPz, Pz); Right posterior (CP4, P4). These ROIs were organized along two topographic dimensions: Anteriority (anterior vs. posterior) and Laterality (left, midline, right).

The ERP data were analyzed with respect to two experimental factors—Structure (Nonisland vs. Island) and QP type (Wh vs. Polar)—at each critical region. For each region, repeated-measures ANOVAs were conducted with Structure, QP, Anteriority, Laterality, and their interactions as within-subject factors. Analyses focused on mean amplitudes in the 350–500 ms (N400), 550–700 ms (P600), 700–850 ms time windows. The 350–550 ms window was used to capture N400 effects, which are classically associated with semantic integration and expectancy-based processing (Kutas and Hillyard, 1980, 1984; Kutas and Federmeier, 2011). The 550–700 ms window was selected to examine P600 effects, typically linked to syntactic reanalysis, structural revision, and increased integration costs (Osterhout and Holcomb, 1992; Kaan and Swaab, 2003). An additional 700–850 ms window was included to explore later sustained positivity/negativity at the QP region, which may reflect extended reanalysis or controlled processing mechanisms.

All statistical analyses were conducted in R (version 4.0.2) using the *afex* package (Singmann et al., 2021), which computes Type III sums of squares with the Kenward–Roger approximation for degrees of freedom. The subject was treated as a random factor in all models.

### 3.6 Results

#### 3.6.1 Behavioral results

In the yes/no comprehension task administered during the ERP experiment, participants showed high overall accuracy, with performance varying across conditions. The [Nonisland–Wh]–FA condition yielded a higher proportion of correct responses (98.7%) than the [Island–Wh]–FA condition (89.3%). In the polar conditions, the [Island–Polar]–PPA condition (90.0%) showed higher accuracy than the [Nonisland–Polar]–PPA condition (83.6%).

Comprehension accuracy was analyzed using logistic linear mixed-effects models implemented in R (version 4.0.2) with the *glmer* function from the *lme4* package (Bates et al., 2015). Structure (Island vs. Nonisland) and Response type (FA vs. PPA) were included as fixed effects, with random intercepts for participants and items. The model revealed a significant main effect of Structure (Estimate = 2.44, SE = 0.36, z = 6.75, *p* < 0.001), indicating that sentences in the Non-island condition were more likely to elicit correct responses than those in the Island condition. Importantly, this effect was qualified by a significant Structure × Response interaction (Estimate = −2.90, SE = 0.24, z value = −11.84, *p* < 0.001), suggesting that the Non-island advantage was substantially diminished in the PPA condition.

Follow-up pairwise comparisons further clarified these patterns. Significant effects of Structure were observed within the FA condition (z = 2.43, *p* < 0.001) and within PPA conditions (z = −3.97, *p* < 0.001). Additionally, a significant effect of Response type was found within the Nonisland condition (z = 8.53, *p* < 0.001), whereas no reliable effect was observed within the Island condition.

#### 3.6.2 Results of the ERP analysis for wh-dependency scope

We analyzed neural responses within the wh-dependency by focusing on two critical regions: the intermediate region (i.e., embedded verbal complex with the embedded complementizer) and the sentence-final QP region. ERP amplitudes were extracted for these regions and analyzed for these regions to track how dependency resolution unfolds across conditions. This approach enabled us to determine whether processing difficulty or integration/feature-matching cost emerges locally at the indeterminate site or later, at the point where the QP is encountered.

##### 3.6.2.1 Results of ERPs at the intermediate region

**Figure 4:**
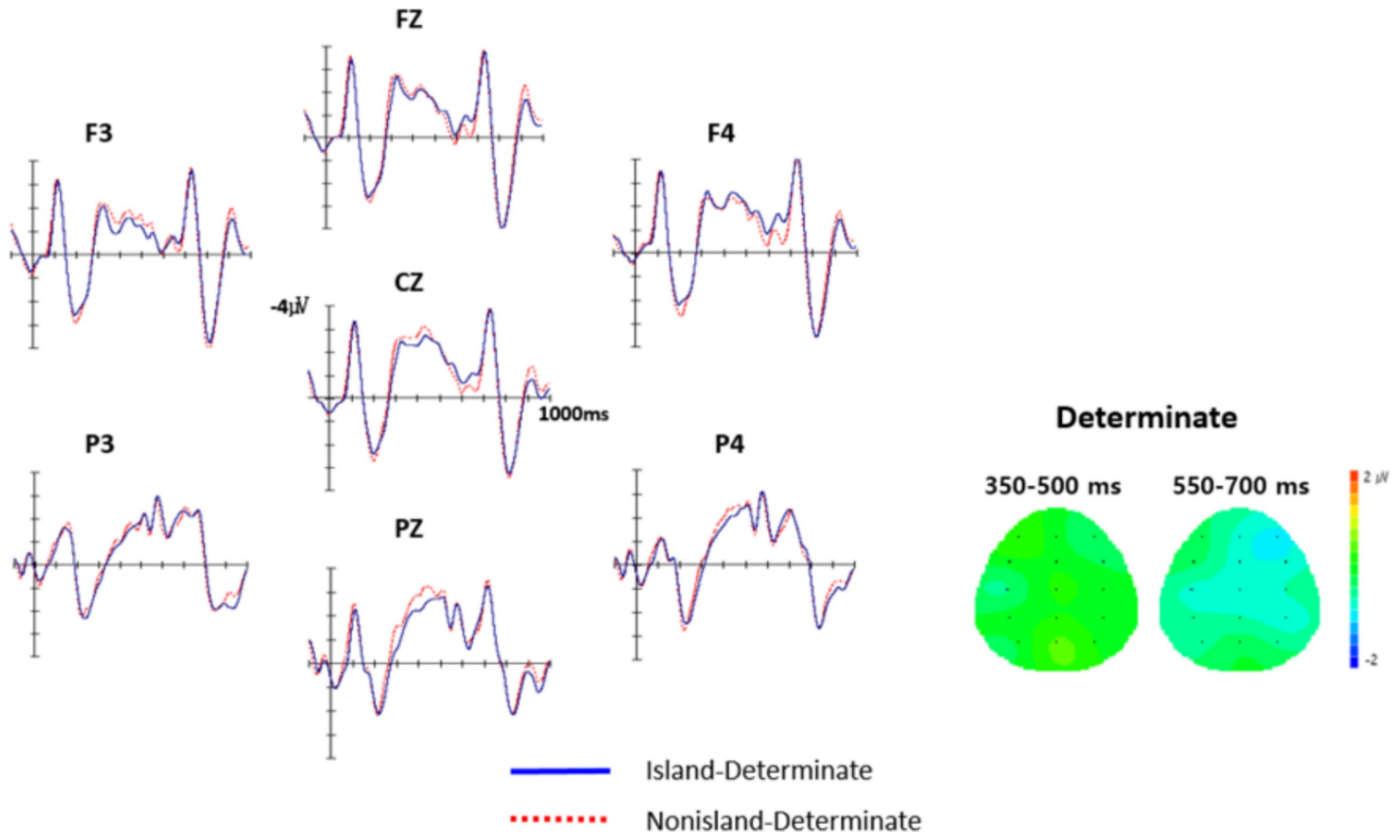
The grand average ERP responses to the determinate region (−*tako*/-*nunci*). The onset of each critical region is indicated by the vertical bar. Each interval represents 100 ms of activity. The negative voltage is plotted upward.

Figure 4 displays the grand-average waveforms for the Nonisland indeterminate and Island indeterminate conditions, along with their corresponding topographic distributions at the critical complementizer region (−*tako*/-*nunci*). Visual inspection of the waveforms indicates that, in the Island condition (e.g., −*nunci*), the ERP response exhibited slightly greater negativity around 600 ms in the right anterior regions compared to the Nonisland condition.

Table 6 presents the results of the repeated-measures ANOVA comparing the Island and Nonisland conditions. The overall analysis revealed no significant main effect of Structure across time intervals. However, a significant main effect of Anteriority was observed in the 550–700 ms interval, *F*(1, 23) = 11.23, *p* < 0.01. In addition, Laterality showed significant effects in both the 350–500 ms interval [*F*(1, 23) = 27.64, *p* < 0.001] and the 550–700 ms interval [*F*(1, 23) = 3.44, *p* < 0.05]. These results motivated a more fine-grained region of interest (ROI) analysis.

**Table 6:** Summary of ANOVA *F*-value at the intermediate region.

Factors	350–500 ms	550–700 ms
*Overall* ANOVA		
(Intercept)	29.25***	17.92***
Island	–	–
Anteriority	–	11.23**
Laterality	27.64***	3.44*

^✝^*p* < 0.1; *p < 0.05; **p < 0.01; ***p < 0.001.

The ROI analysis revealed no significant effects in any region during the 350–500 ms interval. In contrast, during the 550–700 ms interval, a marginally significant effect of Structure emerged at the right anterior sites [*F*(1, 23) = 3.82, *p* = 0.06], reflecting a trend toward greater negativity for the Island condition relative to the Nonisland condition.

##### 3.6.2.2 Results of ERPs at the QP region

**Figure 5:**
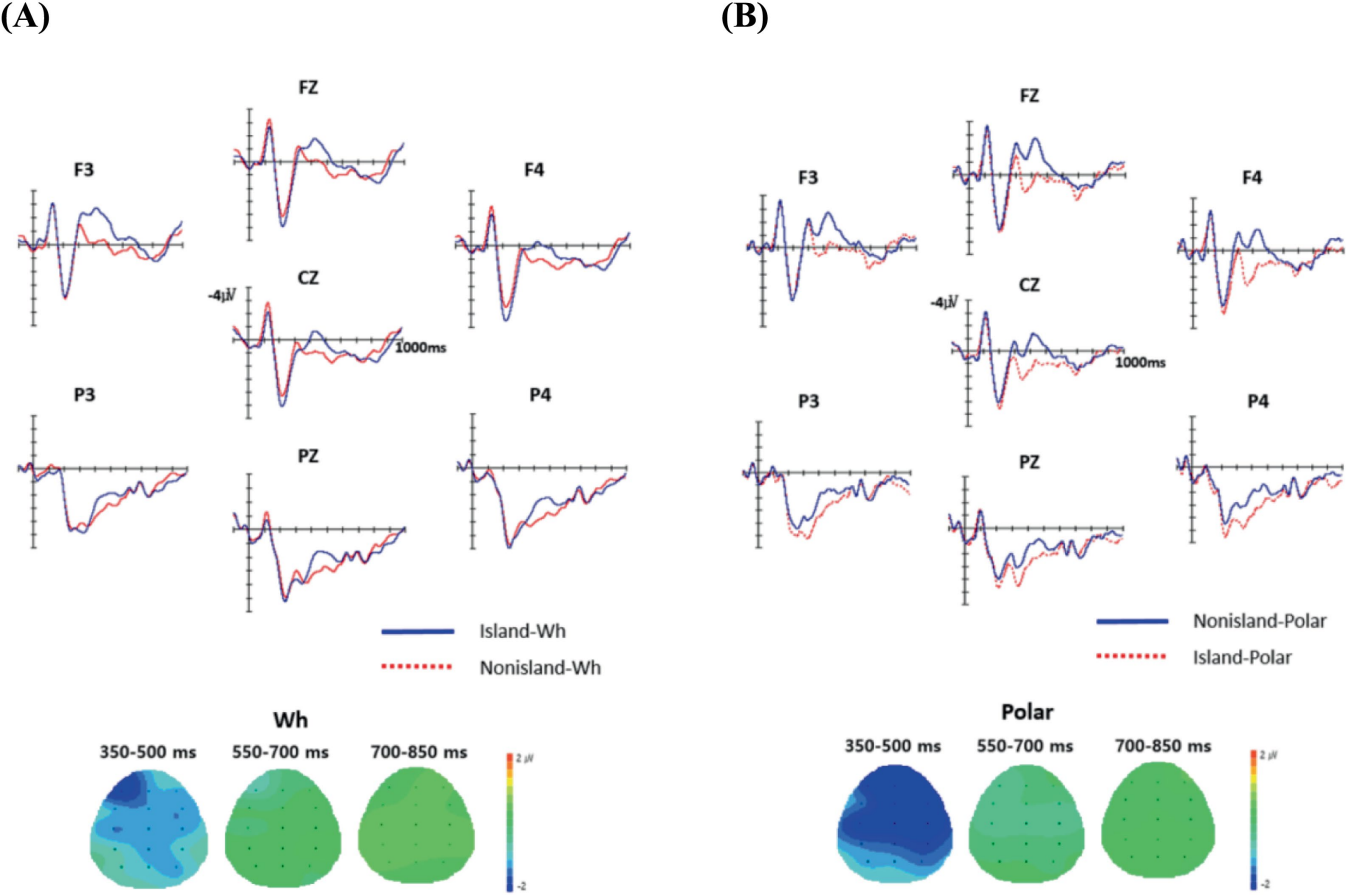
The grand average ERP responses to the Q-particle regions in the four conditions. The onset of each critical region is indicated by the vertical bar. Each interval represents 100 ms of activity. The negative voltage is plotted upward. The first group (5A) of grand waveforms is for the Wh conditions (−*no*) and the second (5B) is for the Polar conditions (−*na*).

Figure 5 presents the grand-average waveforms for the four experimental conditions defined by the interaction of Structure (Nonisland vs. Island) and QP (Wh vs. Polar), along with their corresponding topographic distributions at the critical regions (*alko iss-no* and *alko iss-na*). Visual inspection of the waveforms indicates that in the Island–Wh condition (e.g., *alko iss-no*), a broad negativity emerged around 300 ms, maximal over the left-anterior scalp, compared to the Nonisland–Wh condition. The timing and scalp distribution of this effect are consistent with an N400-like response, typically linked to morpho-syntactic or semantic integration difficulty, expectancy violation, or increased working-memory demands. Additionally, the Island–Wh condition elicited a later posterior positivity in the 700–850 ms interval, consistent with a P600, which is commonly associated with syntactic reanalysis or structural integration. In contrast, the Nonisland–Wh condition showed an earlier positivity (550–700 ms), also suggestive of integration or reanalysis processes.

In the Nonisland–Polar condition (e.g., *alko iss-na*), a robust, broadly distributed negativity appeared around 300 ms, largest over the right-anterior scalp, relative to the Island–Polar condition. This right-anterior N400-like effect (RAN) indicates increased processing difficulty, likely associated with resolving a wh-dependency or feature-matching during semantic integration. No reliable differences were observed between the Nonisland–Polar and Island–Polar conditions in the later 550–700 ms or 700–850 ms intervals.

As summarized in Table 7, the overall ANOVA revealed no main effect of Structure, but a significant main effect of QP in the 700–850 ms window [*F*(1, 23) = 5.82, *p* < 0.05]. Anteriority and Laterality both showed robust effects across all intervals (350–850 ms). Crucially, the Structure × QP interaction was significant in the 300–550 ms interval [*F*(1, 23) = 11.26, *p* < 0.01], driven by enhanced negativities in the Island–Wh and Nonisland–Polar conditions.

**Table 7:** Summary of ANOVA *F*-value at the critical Q-particle.

*Overall* ANOVA	350–500 ms	550–700 ms	700–850 ms
(Intercept)	4.92*	19.19***	42.42***
Structure	–	–	–
QP	–	–	5.82*
Anteriority	39.17***	15.86***	9.58**
Laterality	8.76***	13.11***	5.14**
Structure×QP	11.26**	–	–

Factors	[wh]	[polar]	[wh]	[polar]	[wh]	[polar]
*Pairwise*						
(Intercept)	4.80*	–	26.87***	10.08**	42.17***	26.19***
Structure	4.11*	9.95**	–	–	–	–
Anteriority	35.10***	21.74***	14.16**	7.03*	7.53*	8.67**
Laterality	10.27***	5.70**	16.19***	7.29**	7.22**	–
*Individual ROIs*						
Left Anterior	3.60^†^	6.64*	–	–	–	–
Midline Anterior	–	9.33**	–	–	–	–
Right Anterior	–	11.37**	–	–	–	–
Left Posterior	–	8.96**	–	–	–	–
Midline Posterior	–	8.10**	–	–	–	–
Right Posterior	–	11.73**	–	–	–	–

^†^0.07 < *p* < 0.1; *p < 0.05; **p < 0.01; ***p < 0.001.

Pairwise comparisons corroborated these results. Within Wh conditions, the Island–Wh versus Nonisland–Wh comparison yielded a significant Structure effect in the 350–500 ms interval [*F*(1, 23) = 4.11, *p* < 0.05], reflecting a Left-Anterior Negativity (LAN). Within Polar conditions, the Nonisland–Polar versus Island–Polar contrast revealed a significant Structure effect in the same interval [*F*(1, 23) = 9.95, *p* < 0.01], corresponding to a Right-Anterior Negativity (RAN). Both Anteriority and Laterality showed consistent effects across time windows.

ROI analyses provided further resolution. For Wh conditions, the Island–Wh condition elicited a marginally significant left-anterior effect [*F*(1, 23) = 3.60, *p* = 0.07], consistent with the LAN pattern. For Polar conditions, the Nonisland–Polar condition showed significant Structure effects across all ROIs, most pronounced over right-hemisphere regions [*F*(1, 23) = 11.7, *p*s< 0.05], confirming a robust RAN effect.

##### 3.6.2.3 Results of ERPs at the answer region

**Figure 6:**
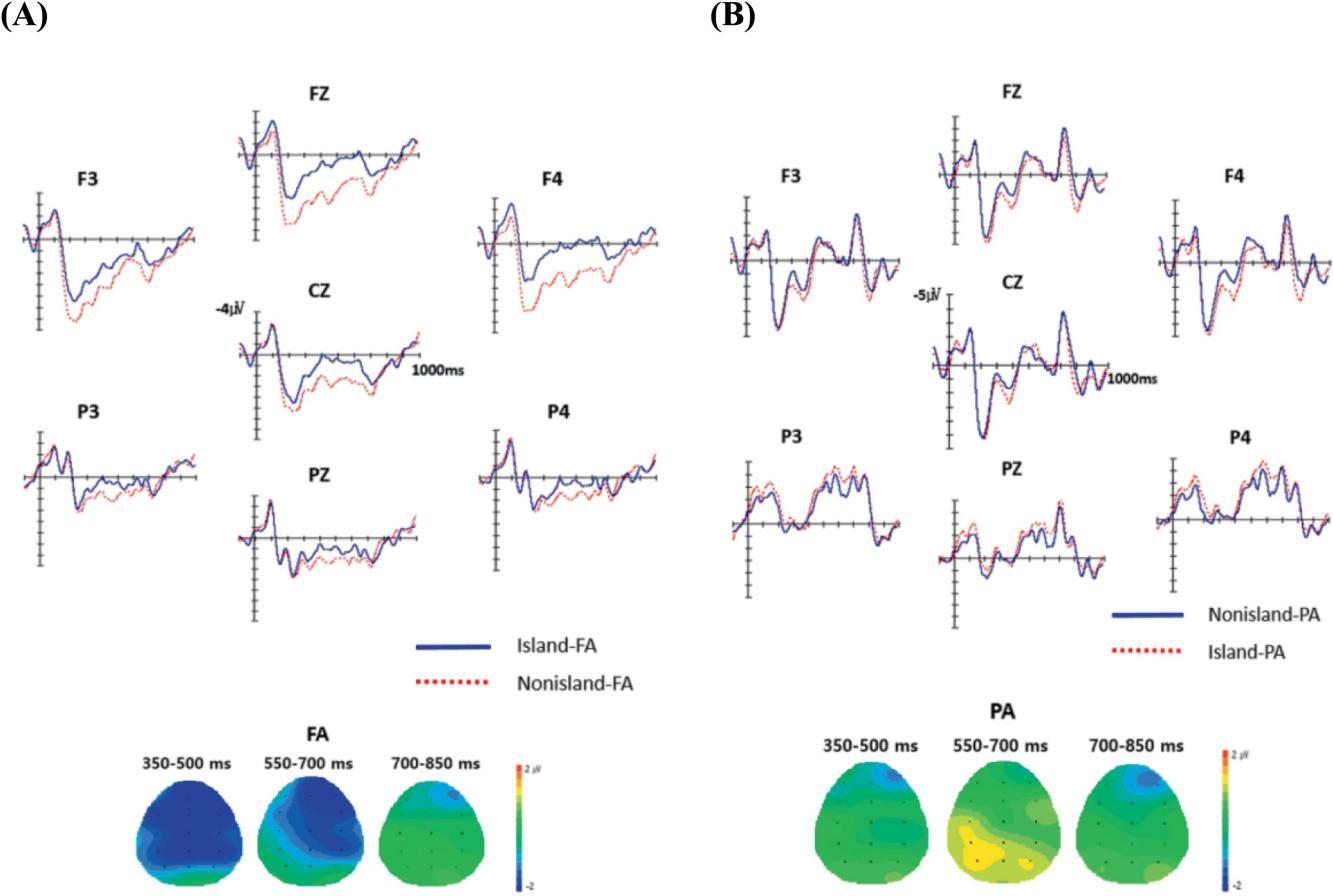
The grand average ERP responses to the answer regions in the four conditions. The onset of each critical region is indicated by the vertical bar. Each interval represents 100 ms of activity. The negative voltage is plotted upward. The first group (6A) of grand waveforms is for the FA conditions (*soselchayk-ul*) and the second (6B) is for the Polar conditions (*ung, alko iss-e*).

Figure 6 presents the grand-average waveforms for the four experimental conditions, each comprising concordant question–answer dialogues [i.e., [+WH] QPs were appropriately followed by fragment answers (FA), whereas polar QPs were followed by positive polarity answers (PPA)]. The corresponding topographic distributions are shown for the critical regions, *soselchayk-u*l and *ung, alko iss-e*. Visual inspection revealed that in the [Island–Wh]-FA condition (e.g., *soselchayk-ul*), a broad negativity emerged around 300 ms and persisted as a long-lasting negative-going wave over anterior scalp regions, relative to the [Nonisland–Wh]-FA condition. This extended anterior negativity (EXAN) is typically interpreted as reflecting the maintenance of a wh-filler in working memory.

In contrast, comparison of the [Nonisland–Polar]-PPA and [Island–Polar]-PPA conditions revealed a positivity in the left-posterior region during the 550–700 ms interval. This P600 effect is generally associated with difficulty in structural/syntactic reanalysis, revision, or repair.^4^

As summarized in Table 8, the overall repeated-measures ANOVA revealed a marginal main effect of Structure–QP in the 350–500 ms interval [*F*(1, 23) = 4.08, *p* = 0.055] and a significant main effect of Response in both the 350–500 ms [*F*(1, 23) = 4.19, *p* = 0.05] and 550–700 ms intervals [*F*(1, 23) = 15.83, *p* < 0.001], and 700–850 ms intervals [*F*(1, 23) = 14.06, *p* < 0.01]. Anteriority and Laterality yielded consistent significant effects across intervals, and critically, the Structure–QP × Response interaction reached significance in the 550–700 ms window [*F*(1, 23) = 4.83, *p* < 0.05], driven by the negativity in the [Island–Wh]-FA condition and the positivity in the [Nonisland–Polar]-PPA condition.

**Table 8:** Summary of ANOVA *F*-value at the critical answer.

Factors	350–500 ms	550–700 ms	700–850 ms
*Overall* ANOVA			
(Intercept)	4.84*	–	–
Structure	4.08^†^	–	–
Response	4.19*	15.83***	14.06**
Anteriority	14.08**	23.13***	22.18***
Laterality	5.14**	11.35***	13.14***
Structure × Response	–	4.83*	–

Factors	FA	PA	FA	PA	FA	PA
*Pairwise*						
(Intercept)	5.77*	–	6.86*	15.44***	10.31**	7.24*
Structure	5.18*	–	5.47*	–	–	–
Anteriority	–	15.34***	–	69.34***	6.72*	22.27***
Laterality	5.09*	–	4.30*	9.39***	12.77***	5.99**
*Individual ROIs*						
Left Anterior	5.67*	–	4.59*	–	–	–
Midline Anterior	5.62*	–	6.42*	–	–	–
Right Anterior	5.48*	–	7.46*	–	–	–
Left Posterior	4.11^†^	–	–	3.16^†^	–	–
Midline Posterior	4.66*	–	4.44*	–	–	–
Right Posterior	–	–	–	–	–	–

^†^0.08 < *p* < 0.1; *p < 0.05; **p < 0.01; ***p < 0.001.

Pairwise comparisons further clarified these effects. Within FA conditions, the [Island–Wh]-FA contrast produced significant Structure–QP effects in both the 350–500 ms [*F*(1, 23) = 5.18, *p* < 0.05] and 550–700 ms intervals [*F*(1, 23) = 5.47, *p* < 0.05], reflecting a robust EXAN pattern. Laterality effects were also significant across intervals. Within PPA conditions, no significant effect of Structure–QP was found, although Anteriority and Laterality continued to show reliable main effects.

A more fine-grained region-of-interest (ROI) analysis confirmed these scalp distributions. For FA conditions, the [Island–Wh]-FA condition elicited significant Structure–QP effects predominantly over anterior sites in both the 350–500 ms [LA: *F*(1, 23) = 5.67, *p* < 0.05; MA: *F*(1, 23) = 5.62, *p* < 0.05; RA: *F*(1, 23) = 5.48, *p* < 0.05) and 550–700 ms intervals (LA: *F*(1, 23) = 4.59, *p* < 0.05; MA: *F*(1, 23) = 6.42, *p* < 0.05; RA: *F*(1, 23) = 7.46, *p* < 0.05], consistent with the EXAN effect. In contrast, for PPA conditions, the [Nonisland–Polar]-PPA condition showed a marginally significant effect of Structure–QP at the left-posterior (LP) region during the 550–700 ms interval [*F*(1, 23) = 3.16, *p* = 0.08], indicative of a P600 component.

##### 3.6.2.4 Anterior ERP responses to failed wh-dependency

**Figure 7:**
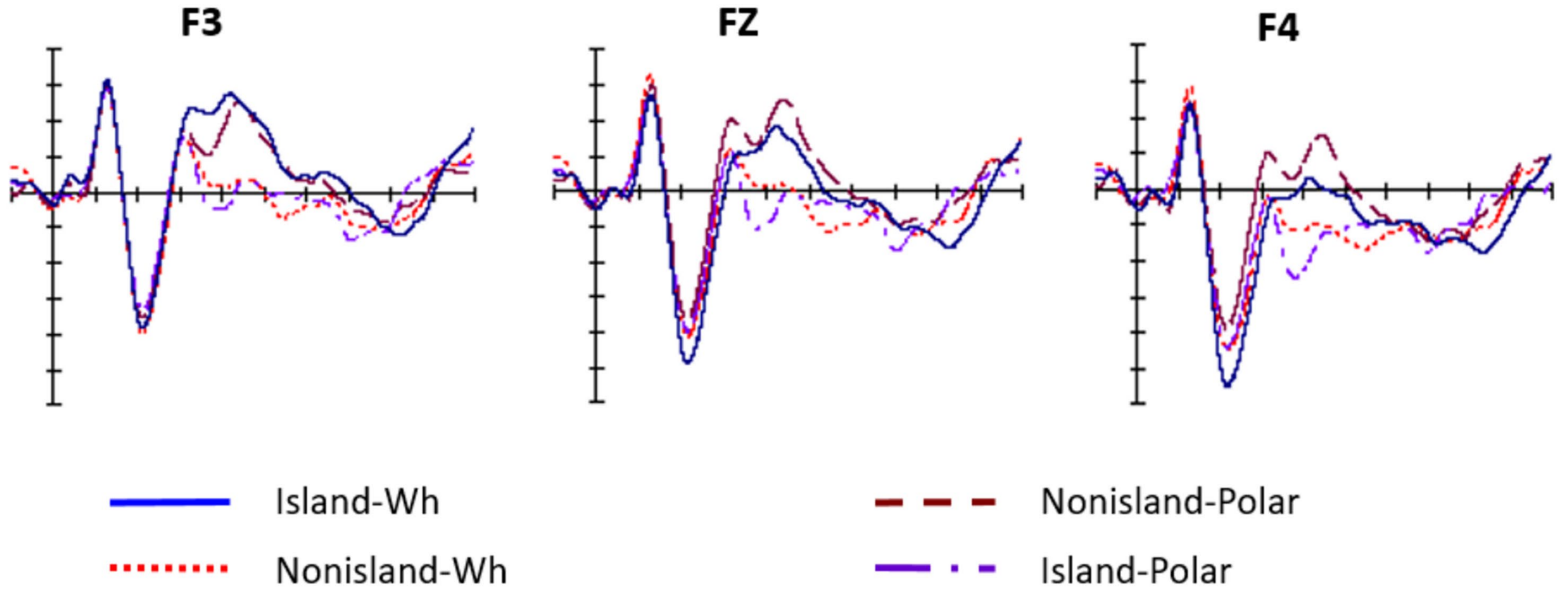
Anterior ERP responses to QP regions for the four conditions. The first group of grand waveforms is for the QP region in the four conditions (*−no or -na*).

**Figure 8:**
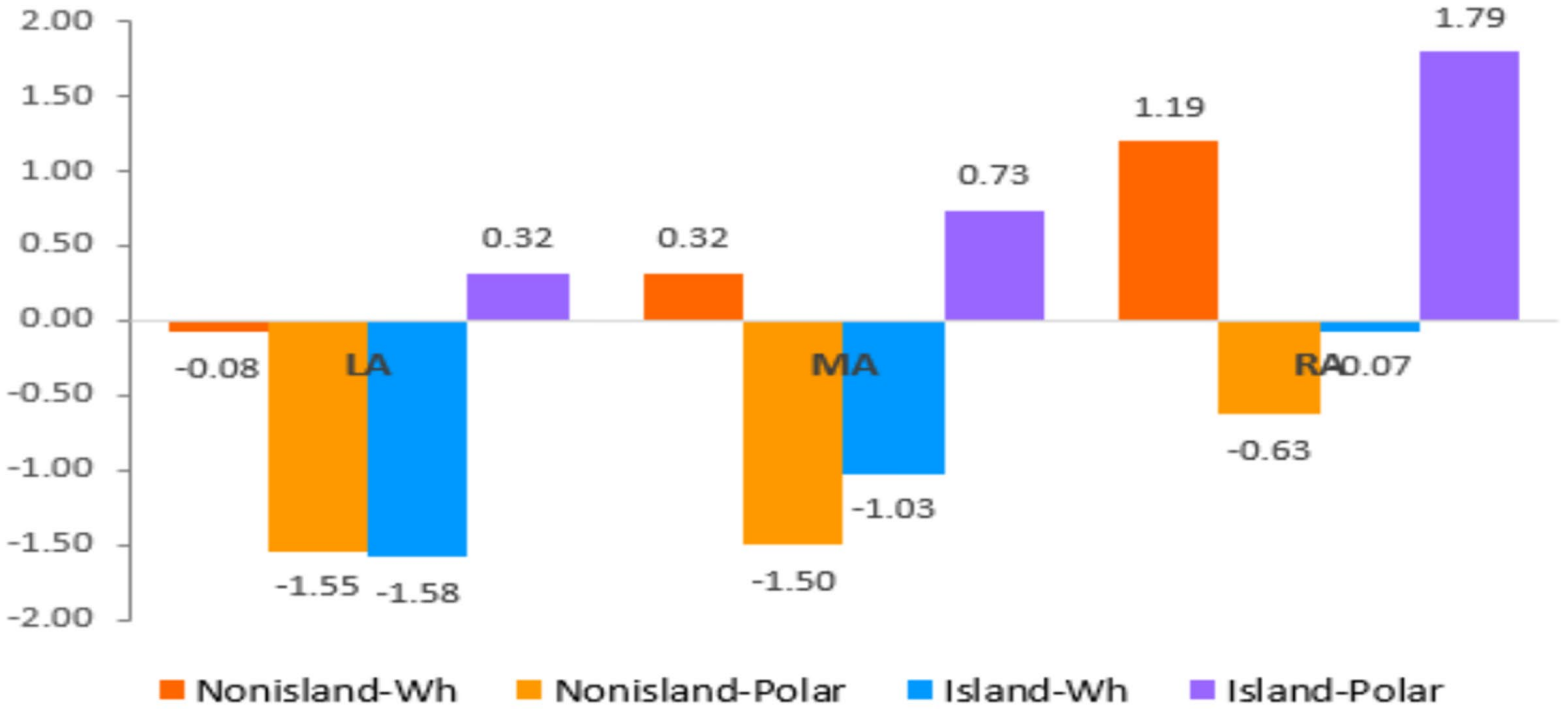
The mean amplitudes for three regions of interest: LA, MA, and RA.

At the QP region, the omnibus analysis revealed significant main effects of Anteriority and Laterality, indicating that ERP responses varied across scalp regions, with effects most prominent over frontal areas and showing hemispheric asymmetries. As illustrated in Figures 7, 8, visual inspection revealed an early divergence between conditions at the QP region, followed by a later convergence over anterior sites. This pattern suggests that the parser initially distinguishes between island and non-island structures, irrespective of the presence or absence of the embedded declarative or interrogative complementizer, yielding condition-specific anterior negativity. As dependency resolution proceeds, these differences diminish, leading to convergent ERP responses across conditions. Such convergence implies that although processing costs differ during the critical licensing phase, the resulting representations are ultimately stabilized comparably across sentence types.

To further examine these effects, mean amplitudes were compared across the left, midline, and right anterior regions for all four experimental conditions. The Island–Wh condition (−1.58 μV) elicited the strongest negativity over left anterior electrodes, whereas the Nonisland–Polar condition (−0.63 μV) exhibited a right-lateralized distribution, predominantly centered over the midline anterior regions. These topographic differences suggest that processing failed Wh–QP dependencies engages distinct frontal neural networks depending on structural constraints and QP type, with lateralization reflecting differential recruitment of cognitive mechanisms such as morpho-syntactic feature maintenance and working-memory demands.

### 3.7 Discussion

We now turn to the ERP results from Experiment 2, which focused on two primary factors: (i) Structure (Island vs. Non-Island) and (ii) QP ([+WH] vs. Polar). ERPs were measured at two critical regions for wh-dependency scope, the embedded complementizer and the matrix QP, to examine how wh-dependency and its interaction with island constraints contribute to the central issue of dependency formation and island sensitivity.

It is important to note that, as shown in the behavioral results presented in the preceding section, indeterminates in KDK are by default interpreted as wh-elements rather than as indefinites. Consequently, they actively anticipate forming a dependency with a [+WH] QP or a [+Q] complementizer. The marginal right-anterior negativity (RAN) observed in the 550–700 ms window at the embedded [+Q] complementizer in the Island condition, relative to the embedded declarative [−Q] complementizer in the Non-island condition, suggests that the embedded wh-indeterminate attempts to establish a local dependency with the embedded [+Q] particle. This pattern is consistent with the Active Filler Strategy (Fodor and Frazier, 1980), according to which the parser, upon encountering a filler (e.g., a wh-phrase), immediately posits a gap (trace) at the earliest grammatically available position. This RAN can be interpreted as a neural correlation of successful integration or feature matching, reflecting the embedded wh-indeterminate’s licit formation of a local dependency with the [+Q] complementizer.

Now we discuss the dependency between the embedded wh-indeterminates and the matrix QPs. In the Nonisland–Polar condition, the embedded wh-indeterminate fails to establish a wh-dependency with the matrix polar QP, whereas in the Island–Polar condition, its local dependency with the embedded [+Q] complementizer makes the use of the matrix polar QP acceptable, resulting in a well-formed configuration. This failure of wh-dependency formation in the Nonisland–Polar condition (II), compared to the Island–Polar condition (IV), elicited a right-anterior negativity (RAN) in the 280–480 ms interval. In other words, in the Nonisland–Polar condition, the wh-indeterminate’s unsuccessful integration with the matrix polar QP gave rise to significant RAN effects over right-anterior scalp regions.

In contrast, attempting to establish a wh-dependency between island-internal wh-indeterminates and the matrix [+WH] QP in the Island–Wh condition (III)—relative to the Nonisland–Wh condition (I), where clause-internal wh-indeterminates in a nonisland/declarative complement can be licitly linked to the matrix [+WH] QP—elicited a left-anterior negativity (LAN) in the 280–480 ms interval. We take this effect to reflect the Q-island constraint induced by the intervening embedded [+Q] complementizer. Importantly, this LAN should not be attributed to retrieval per se, since both (III) and (I) require recovering the relevant wh-indeterminates; rather, it reflects structure-sensitive retrieval difficulty associated with attempting to recover and link wh-indeterminates across a Q-island boundary—that is, reduced cue effectiveness and heightened interference/competition (and possible repair) in the presence of the [+Q] intervener. Thus, while both the Island–Wh (III) and Nonisland–Polar (II) conditions elicited anterior negativities, their underlying sources differ: the LAN in (III) reflects island-related, intervention-driven retrieval/interference (and potential repair) costs during wh-dependency formation, whereas the RAN in (II) reflects failed integration or feature-checking with the matrix polar QP.

These findings closely parallel those reported by Ueno and Kluender (2003, 2009),^5^ who likewise examined the dependency between wh-words and Q-particles in Japanese. Unlike KDK, however, Japanese employs a morphologically identical Q-particle for both polar and wh-questions. Ueno and Kluender found that, relative to main-clause polar questions, main-clause wh-questions elicited a right-lateralized anterior negativity, and that relative to embedded/indirect wh-questions, they elicited an anterior negativity with some rightward bias across a four-word window (300–600, 950–1,250, 1,600–1900, and 2,250–2,550 ms) spanning from the embedded verb to the sentence-final main-clause verb–QP position.

Their main-clause and embedded/indirect wh-questions correspond to our Conditions I and IV, respectively, and both their data and ours reveal the same ERP component—anterior negativity—for embedded relative to main-clause wh-questions (disregarding the later extended negativity beyond 950 ms). By contrast, Ueno and Kluender’s comparison between main-clause wh-questions and polar questions differs in nature from our comparison of the Nonisland–Polar (II) and Island–Polar (IV) conditions, although both sets of contrasts yield the same ERP signature: a right-anterior negativity (RAN). This correspondence, and its broader implications, will be discussed further in the General Discussion section below.

Finally, we turn to the ERP responses at the answer region, which reflect question–answer concord. Relative to the well-formed [Nonisland–Wh]-FA condition, the illicit [Island–Wh]-FA condition elicited an extended anterior negativity (EXAN) beginning around 300 ms and lasting until approximately 700 ms. Recall that the [Island–Wh] configuration is ill-formed because the embedded wh-indeterminate, having already established a local dependency with the embedded [+Q] complementizer, cannot link to the matrix [+WH] QP. Consequently, the [Island–Wh]-FA condition violates question–answer concord.

One possible repair mechanism involves re-linking the embedded wh-indeterminate to the distant matrix QP, an operation that incurs a locality-violation cost. We interpret the observed EXAN as the neural correlate of this repair process: to satisfy question–answer concord, the parser must retrieve the embedded wh-indeterminate from the island domain and associate it with the non-local matrix [+WH] QP, thereby engaging working-memory resources and producing the observed EXAN effect.

In contrast, the [Nonisland–Polar]-PPA condition elicited a P600 effect (550–700 ms) relative to the well-formed [Island–Polar]-PPA condition. The [Nonisland–Polar] configuration is ill-formed because the embedded indeterminate, interpreted by default as a wh-element, cannot establish a dependency with the matrix polar QP. Consequently, the [Nonisland–Polar]-PPA condition fails to achieve question–answer concord, unlike the [Island–Polar]-PPA condition. We interpret the observed P600 as reflecting structural integration difficulty and the engagement of reanalysis and repair mechanisms triggered when the linguistic input violates the expected syntactic or discourse structure.

## 4 General discussion

### 4.1 Wh-indeterminates’ failure in matching with the [+WH] QP: right anterior negativity

The right-anterior negativity (RAN) observed at the matrix QP in the Nonisland–Polar condition, relative to the Island–Polar condition of Experiment 2, is interpreted as reflecting the failure of the embedded wh-indeterminate (or wh-in-situ) to integrate with the polar status of the matrix QP. Alternatively, as one reviewer suggests, the RAN may reflect not only integration failure but also subsequent reanalysis or revision. Although the parser initially interprets the indeterminate as [+WH], upon encountering the matrix polar QP it reanalyzes and revises the element as a [–WH] indefinite. This RAN component is distinct in origin from the similarly right-lateralized anterior negativity reported by Ueno and Kluender (2009) in Japanese, which was elicited within a four-word window spanning the embedded wh-in-situ and its corresponding matrix QP. It is also functionally and temporally differentiated from the combination of sustained and/or phasic LAN and P600 effects observed in wh-questions of wh-fronting languages such as English (Phillips et al., 2005) and German (Fiebach et al., 2001, 2002a, 2002b; Felser et al., 2003).

In the psycholinguistic literature, the displaced wh-element is commonly referred to as a filler, and its base-generated position as a gap, the two forming a dependency essential for sentence interpretation. The filler must be assigned its grammatical function and thematic role by associating with its gap (cf. Fodor, 1989). The processing of such filler–gap dependencies has been consistently linked to the left-anterior negativity (LAN), which may appear in either phasic or sustained form. In addition to its association with phrase-structure or morpho-syntactic violations (Neville et al., 1991; Coulson et al., 1998; Martín-Loeches et al., 2005; see Vos et al., 2001 for review), the LAN is widely taken to reflect working-memory demands incurred when the parser must maintain a displaced filler and retrieve it for integration at the gap site (Kluender and Kutas, 1993a, 1993b; King and Kutas, 1995; Müller et al., 1997; Kluender and Münte, 1998).

For instance, Kluender and Kutas (1993a,1993b) reported phasic LAN effects immediately following both the filler and the gap in English object wh-questions, interpreting these effects as neural correlates of filler storage and retrieval in working memory. Similarly, King and Kutas (1995) observed a sustained frontal negativity between the filler and the gap, followed by a phasic LAN after the gap, during the processing of English object relative clauses.

Subsequent work, however, has identified P600 effects rather than LAN at the gap position. Kaan et al. (2000), for example, found P600 effects preceding the gap in wh-questions compared to yes/no questions, interpreting them as reflecting syntactic integration difficulty associated with linking the filler to the evolving parse (see also Gouvea et al., 2010). Other studies have reported combined sustained/phasic LAN and P600 effects in wh-questions of English (Phillips et al., 2005) and German (Fiebach et al., 2001, 2002a, 2002b; Felser et al., 2003), as well as in scrambled wh-constructions in Japanese (Ueno and Kluender, 2003), indicating that both memory-based and reanalysis-related processes may co-occur during the resolution of wh-dependencies.

Recall that in Experiment 2, the comparison between the Nonisland–Polar and Island–Polar conditions revealed a right-anterior negativity (RAN) at the matrix QP. This component differs in origin from the similarly right-lateralized negativity reported by Ueno and Kluender (2009) in Japanese, which was elicited across a four-word window spanning the embedded wh-in-situ and the corresponding matrix QP. Crucially, unlike in our study, RAN in their experiment did not occur at the termination of the wh–QP dependency. Furthermore, the RAN observed here is distinct from the combined sustained and/or phasic LAN and P600 effects typically found in wh-fronting languages such as English (Phillips et al., 2005) and German (Fiebach et al., 2001, 2002a, 2002b; Felser et al., 2003).

As Ueno and Kluender (2009) convincingly argue, wh-in-situ languages differ fundamentally from wh-fronting languages in that no syntactic displacement or retrieval operation is required to associate wh-phrases with their corresponding Q-particles. Consequently, the RAN observed in Experiment 2 cannot be attributed to the retrieval or syntactic integration of a displaced wh-element at a gap site, as would be the case in wh-fronting languages. Instead, we propose that the RAN at the matrix QP in the Nonisland–Polar condition—relative to the Island–Polar condition—reflects the failure of the embedded wh-indeterminate (wh-in-situ) to match with the polar status of the matrix QP.

In the theoretical literature on wh-in-situ languages, this dependency has been analyzed as a process of Q-binding (e.g., Suh, 1987; Chung, 1996, among others). In KDK, this process is particularly revealing because the dialect morpho-syntactically distinguishes between two types of QPs ([+WH] and [–WH] polar), rendering the matching operation syntactically obligatory. This obligatory matching sets KDK apart from Japanese: the retrieval of the [+WH] feature from the embedded indeterminate and its subsequent matching with the matrix QP appears to directly elicit the RAN component.

Importantly, while this matching process bears some resemblance to agreement [or Agree as a syntactic operation as in Chomsky’s (1995) sense], it differs in a crucial respect. Whereas agreement typically operates under specifier–head or c-command relations, the matching observed here occurs in a reverse c-command configuration.^6^ We therefore suggest that this complex morpho-syntactic matching mechanism—unique to the wh–Q dependency system of KDK—is responsible for generating the RAN when the embedded wh-indeterminate encounters a feature-incompatible matrix polar QP.

The Right Anterior Negativity (RAN) has been documented across a range of linguistic and nonlinguistic domains, consistently emerging in response to violations of higher-order structural constraints. Yamada and Neville (2007) reported a similar ERP pattern elicited by pronouns that could not be integrated into a coherent verb–argument structure. In their grammaticality judgment task (e.g., *Mommy can cut the meat with her that knife), anomalous demonstrative pronouns (*that*) following a complete verb–argument sequence elicited a right-frontal negativity between 180 and 250 ms after onset, relative to control sentences. Likewise, morphosyntactic violations have been shown to evoke right-lateralized negativities: Osterhout and Nicol (1999) observed such an effect (onset ~300–400 ms) for violations of English tense morphology (e.g., *The cat will not eating the food that Mary leaves them*), while Silva-Pereyra and Carreiras (2007) reported a comparable right negativity for agreement violations in Spanish that disrupted the hierarchical organization of tense, person, and number features [e.g., *Vosotros saltas en el patio* ‘You_[2nd plural]_ jump_[1st singular]_ in the backyard’].

Similarly, Jiang and Zhou (2009) found that in Chinese, violations of lower-level constraints on adjectival *de*-modifiers elicited a left-anterior negativity (LAN), whereas violations of higher-level constraints on adverbial *di*-modifiers produced an RAN accompanied by a right centro-parietal N400 between 300–500 ms after onset.^7^ Interestingly, RAN effects have also been observed beyond language: violations of musical syntax—such as harmonically incongruent chords—elicited comparable right-anterior negativities relative to harmonically appropriate progressions (Koelsch et al., 2001; Koelsch et al., 2005; Koelsch and Siebel, 2005). Magnetoencephalographic evidence further localized this right-lateralized response to Broca’s area and its right-hemisphere homolog (Koelsch et al., 2000). Collectively, these findings converge on the view that RAN reflects violations of higher-level, structurally complex constraints within hierarchical systems, whether linguistic or musical.

In parallel with these previous accounts, the RAN observed in the Nonisland–Polar condition of the present study appears to index the parser’s difficulty in resolving complex structural mismatches within the morpho-syntactic system of KDK. During incremental parsing, the parser must first identify the embedded indeterminate as a wh-element requiring association with a clause-final [+WH] QP to determine its interpretive scope. This expectation is maintained in working memory, while the parser actively searches for a suitable QP in a reverse c-command relation with the wh-element—a process consistent with the Active Filler Strategy (Frazier and Clifton, 1989; Stowe, 1986; cf. de Vincenzi, 1991; Gibson, 1998; Just and Carpenter, 1992). At the point of encountering the matrix QP, the parser evaluates whether the wh-indeterminate successfully matches the QP’s morphosyntactic features.

In the Nonisland–Polar condition, this matching process fails, as the embedded wh-indeterminate is feature-incompatible with the matrix polar QP. The resulting processing conflict, involving the attempted integration of mismatched morpho-syntactic information within a hierarchically constrained dependency, manifests as the RAN observed in this condition. Alternatively, the RAN may reflect not only integration failure but also subsequent reanalysis or revision: although the parser may initially treat the indeterminate as [+WH], encountering the matrix polar QP can trigger a reinterpretation in which the element is revised to a [–WH] indefinite, yielding additional anterior negativity associated with this reanalysis process.

### 4.2 Holding [+WH] feature in working memory: left anterior negativity

A left-anterior negativity (LAN) was observed at the [+WH] QP in the Island–Wh condition relative to the Nonisland–Wh condition. This result from Experiment 2 diverges from the findings of Ueno and Kluender (2009), who investigated comparable Japanese constructions. In Japanese, unlike Korean, the matrix clause does not distinguish between [+WH] and polar QPs. In their study, no phasic LAN, RAN, or P600 effects were observed at the QP where wh-scope was disambiguated. We attribute this difference between the two languages to the morpho-syntactic distinctiveness of Q-particles in KDK, which contrasts sharply with Japanese.

In KDK, the matching operation between the wh-indeterminate and the [+WH] QP is syntactically obligatory, owing to the overt morphological distinction between [+WH] and [–WH] polar QPs. This requirement distinguishes KDK from Japanese, where a single, morphologically neutral Q-particle fulfills both [+WH] and polar functions, making the matching relation optional or semantically mediated rather than syntactically enforced. Consequently, in KDK, the [+WH] feature of the embedded wh-indeterminate must be actively maintained in the parser’s working memory until it can be matched with the matrix [+WH] QP. However, the observed effect need not imply that the embedded wh-indeterminate’s feature is definitively licensed within the embedded clause. Rather, upon encountering the embedded V-COMP[+Q], the parser may provisionally associate the wh-indeterminate with the embedded Q-particle as the closest potential licensor. As the sentence continues and the matrix QP is encountered, this initial dependency may be reanalyzed, with the wh-indeterminate ultimately entering into a dependency with the matrix QP instead. The temporary maintenance and subsequent revision of this provisional dependency impose a processing load, giving rise to a LAN. Crucially, the embedded association thus reflects an intermediate parsing hypothesis rather than a stable syntactic relation. Under this view, the LAN indexes the working-memory cost of maintaining, revising, and competing dependency representations during incremental parsing.^8^

The contrast between KDK and English arises from another key difference—overt wh-movement. In English, the displaced wh-filler must be retrieved and integrated at its gap position, a process known to produce phasic LAN and P600 effects. The LAN has been linked to the retrieval of a wh-filler from working memory (Kluender and Kutas, 1993a, 1993b; King and Kutas, 1995),^9^ while the P600 reflects the syntactic integration of the filler at the gap site (Kaan et al., 2000). By contrast, in KDK, wh-indeterminates remain *in situ* and do not undergo syntactic integration, as confirmed by the absence of P600 effects in our data.

Additional insight into this distinction comes from Kluender and Kutas (1993a) ERP study of wh-island contexts in English. They examined sentences such as *Who could not you decide [who/that you/if you should sing something for __ at the family reunion]*? and reported a LAN between 300 and 500 ms, reflecting either the maintenance of a matrix wh-filler in working memory pending gap assignment, or its retrieval for gap resolution. The interaction of these processes yielded the LAN specifically at the point where the matrix wh-filler was assigned to its embedded clause gap. Moreover, the LAN amplitude patterned similarly to the lexically induced N400 at clause boundaries—largest in wh-clauses, smallest in *that*-clauses, and intermediate in *if*-clauses.

Analogously, Q-island contexts in KDK, relative to declarative embedded clauses, reveal a comparable pattern of processing dynamics. The presence of a LAN in these contexts suggests that an embedded wh-indeterminate is actively maintained and retrieved during incremental parsing so that it can, in principle, be related to the sentence-final [+WH] QP. Crucially, however, the contrast between the Island–Wh and Nonisland–Wh conditions indicates that the LAN cannot be attributed to maintenance/retrieval per se (which is required in both conditions). Rather, the modulation of the LAN at the matrix QP position reflects structure-sensitive retrieval difficulty under Q-island constraints: attempting to recover and link wh-indeterminates across an intervening embedded [+Q] complementizer reduces cue effectiveness and increases interference/competition (and possibly triggers repair) relative to a structurally licit nonisland dependency. Thus, the LAN in KDK is best interpreted as indexing island-related, intervention-driven retrieval/interference (and potential repair) costs involved in resolving wh–QP dependencies.

### 4.3 Processing of KDK wh-questions

Taken together, the findings from Experiments 1 and 2, in conjunction with previous studies, provide converging evidence that KDK wh-questions incur distinct processing costs associated with the dependency between a wh-indeterminate and its associated QP. These costs are reflected both in acceptability patterns and in the ERP components—specifically, the Right Anterior Negativity (RAN) and the Left Anterior Negativity (LAN)—recorded at the matrix QP, where wh-scope is ultimately determined.^10^

We have argued that the RAN reflects a feature-mismatch effect between the matrix polar QP and the [+WH] feature retrieved from the embedded wh-indeterminate (or, alternatively, a syntactic reanalysis or revision effect in which the embedded indeterminate is reanalyzed as a [–WH] indefinite). In contrast, the LAN indexes the working-memory cost associated with retrieving and maintaining the [+WH] feature of a wh-indeterminate located within an embedded Q-island, allowing it to participate in an apparently long-distance matching relation with the matrix [+WH] QP. Furthermore, the Extended Anterior Negativity (EXAN) observed for concordant fragment answers to Island–Wh questions is interpreted as reflecting increased working-memory demand in tracking and verifying whether the [+WH] feature of the island-internal indeterminate successfully matches the matrix [+WH] QP.

Importantly, KDK, as a wh-in-situ language, differs from wh-fronting languages in that the wh-indeterminate itself is not syntactically displaced or integrated with the QP. Instead, because KDK morphologically distinguishes [+WH] and polar QPs, only the [+WH] feature of the indeterminate is retained in the parser’s working memory and later enters into a feature-matching relation with the matrix [+WH] QP.

Building on these observations, we propose the following model of wh-question processing in KDK: the parser identifies the embedded indeterminate as bearing a [+WH] feature, maintains this feature in working memory across the clause boundary, and subsequently retrieves it to establish a syntactically licensed but potentially locality-violating feature match with the matrix [+WH] QP—yielding the observed ERP signatures of RAN, LAN, and EXAN, corresponding to feature mismatch, working-memory maintenance, and integration monitoring, respectively.

Upon encountering an indeterminate in KDK, which is by default construed as a wh-element—as evidenced by ERP responses both at the embedded [+Q] complementizer and at the matrix QP, as well as by the behavioral acceptability ratings—the parser recognizes that this element must ultimately be associated with an appropriate QP to determine its syntactic scope. In processing such dependencies, however, the parser does not maintain the entire wh-indeterminate in working memory. Since wh-indeterminates in KDK remain *in situ* and are locally identifiable in terms of grammatical function and thematic role, only their [+WH] feature is stored and maintained in the parser’s working memory. This sharply contrasts with wh-fronting languages like English and German, where the entire displaced wh-filler must be stored and later retrieved for gap integration.

Following Ueno and Kluender (2009), we assume that maintaining the [+WH] feature across the clause boundary until it can be matched with a corresponding matrix [+WH] QP imposes a working-memory cost comparable to that incurred in managing filler–gap dependencies in wh-fronting languages. This cost is reflected in the LAN observed in our data.

At the scope-marking QP position, when an unresolved [+WH] feature from a wh-indeterminate encounters either an embedded [+Q] complementizer or the matrix [+WH] QP, feature-matching occurs, completing the wh-dependency and thereby establishing the scope of the wh-element. In contrast, when a polar QP encounters an unresolved [+WH] feature, the feature-matching process fails. Given that this matching represents a higher-level morpho-syntactic agreement relation, its failure yields a RAN, reflecting difficulty in integrating mismatched feature configurations.

The processing of Q-island contexts in KDK offers further insight into these dynamics. Consistent with the Active Filler Strategy (Frazier and Clifton, 1989), the parser attempts to resolve dependencies as early as possible. When an embedded indeterminate encounters an embedded Q-particle, its [+WH] feature can be licensed locally, yielding an embedded scope construal. Crucially, when the structure later reaches a matrix [+WH] QP, the parser may nonetheless attempt to relate the embedded wh-indeterminate to the matrix operator. We propose that the difficulty at this point is not a matter of retrieval or working-memory load simpliciter (since long-distance retrieval is also required in Nonisland configurations), but rather reflects structure-sensitive retrieval/interference under island constraints. In particular, the intervening embedded [+Q] complementizer (the Q-island boundary) reduces the effectiveness of retrieval cues and increases interference/competition among potential dependency representations, and may additionally induce reanalysis/repair once the dependency is recognized as illicit. The resulting LAN at the matrix [+WH] QP is therefore best interpreted as indexing island-related, intervention-driven retrieval/interference (and possible repair) costs, rather than a failed search for a feature that is “no longer available” in the parser’s workspace.

The cross-linguistic comparison underscores the uniqueness of KDK. Unlike Japanese (Ueno and Kluender, 2009), which employs a morphologically uniform QP for both wh- and polar questions, KDK morphologically distinguishes [+WH] and polar QPs. Consequently, the feature-matching process between the [+WH] feature and QPs is both syntactically obligatory and ERP-sensitive, accounting for the observed asymmetries in neural responses between the two languages. Likewise, KDK differs from English (Kaan et al., 2000; Gouvea et al., 2010): while English involves retrieval and integration of a syntactically displaced filler at a gap position, KDK involves the feature-level retrieval of a wh-indeterminate remaining in situ, with no overt integration step.

When a [+WH] feature from the embedded indeterminate initially construed as a [+WH] element encounters the matrix polar [-WH] QP, proper feature-matching fails, yielding a right-lateralized anterior negativity rather than the left-lateralized effect typically seen in working-memory-driven dependencies. However, as numerous studies have shown (Yamada and Neville, 2007; Osterhout and Nicol, 1999; Silva-Pereyra and Carreiras, 2007; Jiang and Zhou, 2009; Koelsch et al., 2001, 2005), such right anterior negativities are not unique to wh-dependencies; they are elicited by a range of high-level structural violations, including morpho-syntactic and even nonlinguistic (e.g., musical) anomalies. Thus, it is plausible that the RAN in KDK arises from shared neural mechanisms responsible for processing structural incongruities, rather than from a language-specific computation.

In summary, the behavioral and ERP evidence from KDK wh–QP dependencies parallels, in functional terms, the processing patterns reported for filler–gap dependencies in wh-fronting languages. Crucially, the KDK parser attempts to associate a wh-indeterminate with a QP as early as possible, consistent with incremental models of sentence processing. This finding is consistent not only with the Active Filler Strategy (Frazier and Clifton, 1989) but also with related parsing models such as the First-Resort Principle, which states that the parser initially adopts the simplest, most frequent, or default structural attachment for any new input before considering more complex alternatives (Fodor, 1978; Garnsey et al., 1989), and the Minimal Chain Principle, which holds that the parser avoids creating chains (links between a moved element and its trace) unless necessary and, when required, prefers the shortest possible chain (de Vincenzi, 1991). All of these principles propose that the parser attempts to resolve dependencies at the earliest structurally available position. Broadly speaking, the KDK data support incremental processing models (e.g., Inoue and Fodor, 1995) over head-driven accounts (e.g., Pritchett, 1992), demonstrating that even in wh-in-situ languages, wh-dependency formation proceeds predictively and proactively, guided by real-time feature retrieval and matching mechanisms.

## 5 Conclusion

This study demonstrates that wh-in-situ dependency formation in Kyengsang Dialect Korean (KDK) relies on predictive, feature-driven parsing mechanisms that are sensitive to morpho-syntactic agreement and locality. Across two experiments, KDK comprehenders systematically linked wh-indeterminates to sentence-final question particles (QPs) encoding content vs. polar interrogatives, showing that the parser evaluates QP–wh compatibility even without overt wh-movement.

The acceptability results showed that well-formedness depended jointly on feature matching and structural accessibility: configurations that misaligned a wh-indeterminate’s [+WH] feature with a polar QP were reliably penalized, and this penalty was amplified when the putative dependency crossed an island boundary. These interactions support the view that dependency resolution in KDK is not a purely interpretive, post-hoc process, but is modulated by morpho-syntactic licensing conditions and constrained by locality.

The ERP data further clarified the temporal organization of these computations. A Right Anterior Negativity (RAN) emerged for illicit feature combinations, consistent with early sensitivity to [+WH]–QP mismatch. A Left Anterior Negativity (LAN) indexed increased working-memory costs when the [+WH] feature had to be maintained or retrieved across an island. An Extended Anterior Negativity (EXAN) appeared under question–answer discord, suggesting continued monitoring of feature correspondence during integration.

These findings provide converging evidence that KDK speakers maintain and retrieve a [+WH] feature representation in order to establish syntactically licensed dependencies with sentence-final QPs. Importantly, the results show that dependency resolution is guided by both feature compatibility and structural accessibility: mismatches between a wh-indeterminate and a polar QP trigger early feature-evaluation costs (RAN), while attempts to maintain a potential dependency across an embedded [+Q] environment increase working-memory demands (LAN). Together, these results demonstrate that wh-in-situ interpretation in KDK proceeds through incremental dependency formation that is sensitive to both morpho-syntactic licensing and locality constraints, rather than through purely interpretive scope assignment at the sentence level.

## Data Availability

The datasets and results for this study can be found in the OSF: https://osf.io/3pk7e/overview?view_only=e6e48998c7614163b8fa1aa44fedfe48.

## Footnotes

1 Previous studies have offered several influential accounts of wh-island effects. Ross (1967) was the first to identify and formulate the wh-island constraint, observing that wh-extraction out of an embedded wh-clause is systematically degraded. Chomsky et al. (1973, 1977) derived the constraint from Subjacency, proposing that wh-movement cannot cross more than one bounding node (S/NP) at a time; when the embedded [Spec, CP] is occupied, the usual escape hatch is unavailable, forcing an illicit long-distance dependency. Huang (1982) refined this view by highlighting an argument–adjunct asymmetry, arguing that in wh-in-situ languages, dependencies are formed at LF: arguments (e.g., what) may sometimes escape wh-islands because they are lexically governed, whereas adjuncts (e.g., why, how) must satisfy antecedent government and therefore incur strong ECP violations when extracted. Building on this line of work, Rizzi (1990) reanalyzed wh-island effects in terms of Relativized Minimality, proposing that an intervening [+Q] operator in the embedded [Spec, CP] blocks dependency formation; adjuncts, which require strictly local chains, are fully blocked, while referential arguments may occasionally circumvent the intervener via binding, yielding weaker island effects.

2 All experimental dataset and results for this study can be found in: https://osf.io/3pk7e/overview?view_only=e6e48998c7614163b8fa1aa44fedfe48

3 One concern is that offline tasks allow time for “repair,” potentially masking initial processing difficulties. However, our effects are robust. First, successful repair would have yielded uniform acceptability; instead, we observed significant reductions. Second, comparing the low scores of Conditions II (2.37) and III (2.01) against grammatical counterparts (I: 4.00, IV: 4.45) confirms these structural violations are too severe to be recovered through reanalysis. Our Likert-scale data thus captures valuable gradient information about these constructions’ perceived naturalness.

4 The three notions used in this paper—structural/syntactic reanalysis, revision, and repair—follow the account in Kaan et al. (2000). Structural or syntactic reanalysis refers to the parser’s general process of revising an initially assigned syntactic structure when later input conflicts with it. Structural or syntactic revision more specifically denotes the transition from the initial representation to a new structural analysis compatible with the incoming input. Structural or syntactic repair, by contrast, refers to the parser’s attempt to accommodate or resolve a syntactic violation by adjusting the structure to satisfy grammatical constraints.

5 There has been ample discussion in the previous ERP literature on the processing of long-distance wh-dependencies in wh-in-situ languages such as Japanese (Nakagome et al., 2001; Ueno and Kluender, 2003, 2009) and Mandarin (Lo and Brennan, 2021), as well as in overtly wh-fronting language such as German and English (Fiebach et al., 2002a, 2002b; Phillips et al., 2005). Since Ueno & Kluender’s works on long-distance wh-dependencies in Japanese are directly relevant to our study of the counterparts in KDK, they serve as references in investigating the latter in the text.

6 The view adopted in this paper that the matching between a wh-in-situ and a QP occurs in a reverse c-command configuration is consistent with the notion of Upward Agree proposed in recent syntactic work. While the Minimalist Program (Chomsky, 1995) assumes Downward Agree, whereby a higher probe searches for a lower goal, Zeijlstra (2012) and Wurmbrand (2012) argue that agreement may instead proceed upward, with a lower element establishing an agreement relation with a higher c-commanding element, as evidenced by phenomena such as negative concord and tense/auxiliary systems.

7 Lower-level constraints involve local syntactic category matching (LAN), while higher-level constraints require integrating modifiers into the broader thematic and event structure, eliciting more complex neural responses like RAN and N400.

8 As an alternative to accounts of wh- or Q-island effects that rely on working memory maintenance, cue-based retrieval models (Vasishth and Lewis, 2006) attribute these effects to similarity-based interference during memory retrieval rather than solely to syntactic constraints. When the parser attempts to retrieve a stored wh-filler using feature cues, an intervening wh-phrase sharing the feature [+wh] creates competition, thereby increasing processing difficulty (Gordon et al., 2001). Psycholinguistic evidence from Mandarin further suggests that wh-in-situ dependencies are processed through comparable retrieval mechanisms: intervening clause boundaries function as distractors and reduce retrieval accuracy (Xiang et al., 2015). Within this perspective, a [+Q] feature on an intervening wh-phrase or complementizer acts as a source of proactive interference; because it matches the parser’s retrieval cues, it disrupts the memory search process and prevents successful retrieval of the intended wh-dependency target. By contrast, Rizzi (1990) treats the [+Q] feature as a structural barrier that syntactically blocks the formation of such dependencies.

9 Reductionist accounts that attribute island effects to working memory (WM) limitations propose that the combined cost of filler storage and structural complexity overwhelms processing resources (Kluender & Kutas, 1993b). However, large-scale studies have found no correlation between individual WM capacity and island sensitivity (Sprouse et al., 2012; Aldosari, 2015; Pañeda et al., 2020). Even speakers with high WM show the same sharp decline in acceptability as those with lower WM. These findings suggest that island effects reflect grammatical constraints rather than processing limits, a conclusion further supported by cross-linguistic evidence (Pham et al., 2020). This does not mean that WM plays no role in sentence processing; rather, it indicates that the core degradation associated with island violations stems from a formal syntactic constraint. While speakers with higher WM may process longer or more complex sentences more efficiently, they still judge island structures as equally unacceptable.

10 The RAN and LAN effects do not represent a divide between syntax and working memory; instead, they reflect the integrated syntactic lifecycle of the wh-dependency at issue. Following the one-system hypothesis (Phillips, 2004), we argue that grammatical constraints and parsing mechanisms are functionally identical. The RAN (Condition I) indexes initial syntactic licensing, while the LAN (Condition III) reflects the real-time structural construction of a non-local link. Within a cue-based retrieval framework (Vasishth and Lewis, 2006), this memory cost is inherently syntactic, as it is triggered by structural similarity-based interference (e.g., the intervening [+Q] feature) during the search for a scope position. Thus, the LAN provides direct evidence for a psychologically real syntactic dependency.
